# Intertwining Operators Beyond the Stark Effect

**DOI:** 10.1007/s00220-026-05600-w

**Published:** 2026-04-04

**Authors:** Luca Fanelli, Xiaoyan Su, Ying Wang, Junyong Zhang, Jiqiang Zheng

**Affiliations:** 1 Ikerbasque, Basque Foundation for Science, Departamento de Matemáticas, Universidad del País Vasco/Euskal Herriko Unibertsitatea, PV/EHU 48940 Leioa, Spain; 2 BCAM - Basque Center for Applied Mathematics, 48009 Bilbao, Spain; 3 Loughborough University, Loughborough, UK; 4 Department of Mathematics, Beijing Institute of Technology, Beijing, 100081 People’s Republic of China; 5 MSU-BIT-SMBU Joint Research Center of Applied Mathematics, Shenzhen MSU-BIT University, Shenzhen, 518172 People’s Republic of China; 6 Institute of Applied Physics and Computational Mathematics and National Key Laboratory of Computational Physics, 100088 Beijing, People’s Republic of China

## Abstract

The main mathematical manifestation of the Stark effect in quantum mechanics is the shift and the formation of clusters of eigenvalues when a spherical Hamiltonian is perturbed by lower order terms. Understanding this mechanism turned out to be fundamental in the description of the large-time asymptotics of the associated Schrödinger groups and can be responsible for the lack of dispersion (Fanelli et al. in Commun Math Phys 324:1033–1067, 2013; in Commun Math Phys 337:1515–1533, 2015; in J Spectr Theory 8:509–521, 2018). Recently, Miao et al. introduced in Miao et al. http://arxiv.org/abs/2405.02531 a family of spectrally projected *intertwining operators*, reminiscent of the Kato’s wave operators, in the case of constant perturbations on the sphere (inverse-square potential), and also proved their boundedness in $L^p$. Our aim is to establish a general framework in which some suitable intertwining operators can be defined also for non constant spherical perturbations in space dimensions 2 and higher, which is highly non trivial. In addition, we investigate the mapping properties between $L^p$-spaces of these operators. In 2D, we prove a complete result, for the Schrödinger Hamiltonian with a (fixed) magnetic potential an electric potential, both scaling critical, allowing us to prove dispersive estimates, uniform resolvent estimates, and $L^p$-bounds of Bochner–Riesz means. In higher dimensions, apart from recovering the example of inverse-square potential, we can conjecture a complete result in presence of some symmetries (zonal potentials), and open some interesting spectral problems concerning the asymptotics of eigenfunctions.

## Introduction

In 1913, Johannes Stark observed a peculiar phenomenon now known as the Stark effect: under the influence of a non-constant external electric field, the energy levels of an atom shift and split into multiple lines, clustering into specific formations [37]. This effect is analogous to the mechanism observed under a static external magnetic field, as first identified by Pieter Zeeman in 1896 [49]. Mathematical evidence studying the spectrum of $-\Delta _{{\mathbb {S}}^{d-1}} + a(\theta )$ of the above effect can be obtained by analyzing the spectrum of the spherical Schrödinger Hamiltonian, $-\Delta _{{\mathbb {S}}^{d-1}}$ when perturbed by a lower-order external force. Due to the significant experimental and theoretical implications, extensive literature has been devoted to the study of the spectrum of operators of the form $-\Delta _{{\mathbb {S}}^{d-1}} + a(\theta )$, where $a:{\mathbb {S}}^{d-1} \rightarrow {\mathbb {R}}$. This type of perturbation typically results in the formation of eigenvalue clusters around the unperturbed energy levels $j(j+d-2)$, with $j \in {\mathbb {N}}$, together with a uniform shift, provided that the potential is non-constant and satisfies certain symmetry requirements. Some pioneering works, such as those by D. Gurarie [27] and by L. Thomas and S. Wassell [40], have established this clustering behavior, with extensive references therein. When $d=2$ (corresponding to a one-dimensional spherical problem), this clustering effect is nearly universal, requiring only minimal regularity conditions on the potentials (see [18, Lemma 2.1] for detailed asymptotics of eigenvalues and eigenfunctions.)

In the past decade, beginning with [16], a deep connection has emerged between these spectral properties and the large-time asymptotics of scaling-invariant Schrödinger groups. Broadly, the structure and separation of high-energy clusters, along with the asymptotic profiles of the associated eigenfunctions, are crucial in analyzing the high-energy band in the time-decay properties of these Schrödinger groups in the full space, as showed in several recent papers (see e.g. [18–22, 24–26, 35]). A canonical model within this context is the Schrödinger Hamiltonian with an inverse-square potential $-\Delta +\frac{a}{|x|^2}$, *a* being a constant. Restricting this model to ${\mathbb {S}}^{d-1}$ results in a constant perturbation of the Laplace-Beltrami operator on the sphere, $-\Delta _{{\mathbb {S}}^{d-1}}+a$. The large-time behavior of the propagator ${e^{it(-\Delta +\frac{a}{|x|^2})}}$, including decay and Strichartz estimates, is now well understood, particularly for dimensions $d=2$ and 3. In this case, the spectral problem is straightforward as the spectrum only sees a uniform shift, without cluster formation. Specifically, the spectrum of $-\Delta _{{\mathbb {S}}^{d-1}}+a$ is given by the numbers $j(j+d-2)+a$, with $j\in {\mathbb {N}}$, and the eigenfunctions are spherical harmonics, with shifted index. This leads to a direct one-to-one correspondence between the unperturbed and perturbed eigenvalues, preserving their order and allowing the definition of some operators that map spectral projections before perturbation to their perturbed counterparts. Miao, Su, and Zheng in [34] introduced such operators, which exhibit an intertwining property that allows to represent the perturbed functional calculus in terms of the unperturbed one. This concept aligns with the classic wave operators introduced by T. Kato, [31], whose $L^p$-boundedness properties have been extensively studied, due to their applications in dispersive estimates and scattering theory, in [11, 13, 14, 42–48] and many other papers.

The objective of this manuscript is to establish a rigorous mathematical foundation for defining similar intertwining operators as those in [34] also in presence of cluster formations. Extending this analysis beyond the Stark effect needs a precise combinatorial analysis of the energy levels and their associated eigenfunctions after the splitting. Once this structure is understood, we propose a general methodology to prove the $L^p$-boundedness of these operators. This involves a novel discrete multiplier result with variable coefficients (Lemma 2.3 below) and detailed decay estimates of oscillatory integrals.

### Mathematical Framework

Let us consider an electromagnetic Schrödinger operator on ${\mathbb {R}}^{d}$, $d \ge 2$, of the form

$$\begin{aligned} {\mathcal {L}}_{{\textbf{A}},a}:=\left( -i\nabla +\frac{{\textbf{A}}(\theta )}{|x|}\right) ^2+\frac{a(\theta )}{|x|^2}, \end{aligned}$$

where $\theta =\frac{x}{|x|}\in {\mathbb {S}}^{d-1}$, $a\in L^{\infty }({\mathbb {S}}^{d-1},{\mathbb {R}})$ and ${\textbf{A}}\in C^1 ({\mathbb {S}}^{d-1},{\mathbb {R}}^d)$ is a transversal vector field,

1.1 $$\begin{aligned} {\textbf{A}}(\theta )\cdot \theta =0\text { for all }\theta \in {\mathbb {S}}^{d-1}. \end{aligned}$$

The differential operator ${\mathcal {L}}_{{\textbf{A}},a}$ acts on complex-valued functions $f\in C^\infty ({\mathbb {R}}^d\setminus \{0\})$ as

$$\begin{aligned} {\mathcal {L}}_{{\textbf{A}},a}f =-\Delta f+\frac{|{\textbf{A}}(\theta )|^2 +a(\theta )-i \operatorname {div}_{{\mathbb {S}}^{d-1}} {{\textbf{A}}(\theta )}}{|x|^2} -2i \frac{{\textbf{A}}(\theta )}{ |x|} \cdot \nabla f, \end{aligned}$$

where $\operatorname {div}_{{\mathbb {S}}^{d-1}} {{\textbf{A}}(\theta )}$ denotes the Riemannian divergence of ${{\textbf{A}}}$ on the unit sphere ${\mathbb {S}}^{d-1}$ endowed with the standard metric.

In spherical coordinates $x=r \theta$, $r=|x|$, $\theta =\frac{x}{|x|}$, ${\mathcal {L}}_{{\textbf{A}},a}$ can be written as

$$\begin{aligned} \begin{aligned} {\mathcal {L}}_{{\textbf{A}},a}=-\partial _r^2-\frac{d-1}{r}\partial _r+\frac{L_{{\textbf{A}},a}}{r^2}, \end{aligned} \end{aligned}$$

where the spherical operator $L_{{\textbf{A}},a}$ is defined by

$$\begin{aligned} L_{{\textbf{A}},a}=(-i\nabla _{{\mathbb {S}}^{d-1}}+{\textbf{A}})^{2}+a(\theta ), \end{aligned}$$

where $\nabla _{{\mathbb {S}}^{d-1}}$ is the spherical gradient on the unit sphere ${\mathbb {S}}^{d-1}$. By standard spectral theoretical arguments, we see that the spectrum of $L_{{\textbf{A}},a}$ is purely discrete, and consists of a diverging sequence of real eigenvalues $\lambda _n({\textbf{A}},a) \uparrow + \infty$ where each eigenvalue is repeated according to its finite multiplicity (see e.g. [23]).

Under the condition that

1.2 $$\begin{aligned} \lambda _1({\textbf{A}},a)\ge -\Big (\frac{d-2}{2}\Big )^2, \end{aligned}$$

the Hamiltonian ${\mathcal {L}}_{{\textbf{A}},a}$ can be understood as the Friedrichs’ extension of the natural quadratic form on $L^2({\mathbb {R}}^d,{\mathbb {C}})$ [31, Theorem VI.2.1], with the form domain

$$\begin{aligned} {\mathcal {D}}({\mathcal {L}}_{{\textbf{A}},a}) =\bigg \{ f\in L^2({\mathbb {R}}^d;{\mathbb {C}}): \int _{{\mathbb {R}}^d} \bigg |\nabla f+i\frac{{\textbf{A}}(\theta )}{|x|}f\bigg |^2+\frac{a(\theta )}{|x|^2}|f|^2 \,\,\textrm{d}x <\infty \bigg \}. \end{aligned}$$

As explained above, the goal of this manuscript is to introduce some intertwining operators which enable us to reduce the functional calculus associated to ${\mathcal {L}}_{{\textbf{A}}, a}$ to the one for the electric-free operator ${\mathcal {L}}_{{\textbf{A}},0}$. Once such an object is available, its mapping properties between $L^p$-spaces will permit us to obtain relevant information about the large-time asymptotics of the Schrödinger group $e^{it{\mathcal {L}}_{{\textbf{A}}, a}}$, as well as the resolvent of ${\mathcal {L}}_{{\textbf{A}}, a}$, by inheriting these properties from ${\mathcal {L}}_{{\textbf{A}}, 0}$.

When $d=2$, a fundamental example of a magnetic field in the above class is the so called *Aharonov–Bohm* potential (hereafter ${{\,\textrm{AB}\,}}$), defined by

Aharonov--Bohm $$\begin{aligned} {{\,\textrm{AB}\,}}(x):=\frac{{\textbf{A}}(\theta )}{|x|}, \qquad {\textbf{A}}(\theta )=\alpha \left( -\frac{x_2}{|x|}, \frac{x_1}{|x|}\right) ,\quad \alpha \in {\mathbb {R}}. \end{aligned}$$

In Aharonov and Bohm’s paper [2], the phenomenon of scattering in regions in which the electromagnetic field is absent was predicted within the framework of Schrödinger dynamics. This phenomenon was later incontrovertibly confirmed by experiments of Tonomura et al. [36]. The ${{\,\textrm{AB}\,}}$ model serves as a 2D idealization of a 3D model involving an infinitely long and thin solenoid carrying an electric current, which confines a magnetic field within its interior. Recently, experimental evidence of 2D quasiparticles, known as *anyons*, which naturally carry on an ${{\,\textrm{AB}\,}}$-type magnetic field [3], has reinforced the significance of this 2D model. Notably, the ${{\,\textrm{AB}\,}}$ Schrödinger Hamiltonian ${\mathcal {L}}_{{{\,\textrm{AB}\,}},0}$ retains the same scaling invariance as the free operator ${\mathcal {L}}_{0,0}$, highlighting the scaling-critical nature of this perturbation. We also mention [1, 5] as standard references about the self-adjoint realization of such Hamiltonian.

In the context of zero-order perturbations, the canonical example in any dimension is the inverse-square potential operator ${\mathcal {L}}_{0,a}$ where ${a\ge -\frac{(d-2)^2}{4}}$ is a constant. Also in this case, we have that ${\mathcal {L}}_{0,a}$ scales as ${\mathcal {L}}_{0,0}$.

Extensive research on such operators has focused on the validity of some families of estimates that characterize the Schrödinger equation as the prototypical dispersive PDE. Burq, Planchon, Stalker, and Tahvildar-Zadeh proved in [7, 8] that the standard Strichartz estimates are valid in the presence of zero-order perturbations with the same scaling as the inverse-square. Later, in [16, 18] it was shown that the decay estimate

$$\begin{aligned} \Vert e^{it{\mathcal {L}}_{{\textbf{A}},a}}\Vert _{L^1({\mathbb {R}}^2)\rightarrow L^\infty ({\mathbb {R}}^2)}\lesssim |t|^{-1}, \end{aligned}$$

which implies Strichartz estimates by standard results, holds generally in 2D. In 3D, this estimate holds for the defocusing inverse-square potential case $a\ge 0$, but fails for the focusing case $-(d-2)^2/4\le a<0$ [16]. The proofs in [16, 18] rely on a detailed analysis of the Stark and Zeeman effects post-perturbation. These results have been later extended to other dispersive operators (see [21, 22, 25, 35] and references therein).

This paper aims to address the spectral structure of operators ${\mathcal {L}}_{{\textbf{A}}, a}$ and ${\mathcal {L}}_{{\textbf{A}},0}$ for fixed ${\textbf{A}}$, by developing a rigorous framework which may also hold in presence of cluster formations for defining intertwining operators under specific spectral conditions. Establishing such operators and bounding them in $L^p$-spaces will require careful spectral analysis, as well as some novel harmonic analytical tools which will come into play in the sequel.

### Spectral Framework

The free spherical operator $L_{0, 0}=-\Delta _{{\mathbb {S}}^{d-1}}$ has purely discrete spectrum, given by the eigenvalues $\eta _{j}^2$ where $\eta _j:=\sqrt{j(j+d-2)}\ge 0$, each one with multiplicity $m_j:=\frac{(d+j-1) !}{(d-1)! j!}=O(j^{d-2})$. Moreover, the usual spherical harmonics are the corresponding eigenfunctions. Having compact inverse, the operators $L_{{\textbf{A}}, 0}$ and $L_{{\textbf{A}}, a}$ also have purely discrete spectrum with eigenvalues accumulating at infinity (see e.g. [16]). The expected spectral picture for $L_{{\textbf{A}},a}$ after the Stark–Zeeman effect suggests to assume from now on that the following conditions hold for $L_{{\textbf{A}}, 0}$ and $L_{{\textbf{A}}, a}$:

#### Assumption 1.1

 

1. **Asymptotics of Clusters.** There exist $\ell \in {\mathbb {N}}$ and a constant $0<C_{{\textbf{A}}}<1/2$, only depending on ${\textbf{A}}$, such that for any $j\ge \ell$, the free eigenvalues $\eta _j^2=j(j+d-2)$ split into clusters of eigenvalues of the operators $L_{{\textbf{A}}, 0}$ and $L_{{\textbf{A}}, a}$, each with exactly $m_j:=\frac{(d+j-1) !}{(d-1)! j!}$ eigenvalues (with possible repetitions), denoted by $\mu _{jk}^2$ and $\nu _{jk}^2$, $k=1,\dots , m_j$, respectively, with the following localization property:
  $$\begin{aligned} \eta _{j} +C_{{\textbf{A}}}-\frac{1}{2}< \mu _{j 1} \le \mu _{j 2} \le \dots \le \mu _{j m_j} \le \eta _{j} +C_{{\textbf{A}}}+ \frac{1}{2}, \\ \eta _{j} +C_{{\textbf{A}}}-\frac{1}{2} < \nu _{j 1} \le \nu _{j 2} \le \dots \le \nu _{j m_j} \le \eta _{j} +C_{{\textbf{A}}}+ \frac{1}{2}. \end{aligned}$$
  The corresponding normalized eigenfunctions are denoted by $e_{jk}$ and $\phi _{jk}$ $( k=1, \dots , m_j )$, respectively, i.e. for all $j \ge \ell ,$ and all $1\le k \le m_j$,
  $$\begin{aligned} L_{{\textbf{A}},0}e_{jk}(\theta )=\mu _{jk}^2e_{jk}(\theta ), \quad \text {and}\quad L_{{\textbf{A}},a}\phi _{jk}(\theta )=\nu _{jk}^2\phi _{jk}(\theta ). \end{aligned}$$
2. **Simultaneous Completion.** There exists $\ell _0$ (possibly zero) depending on $\ell$, a set $\{e_i\}_{i=1,2, \dots , \ell _0}$ of normalized eigenfunctions of $L_{{\textbf{A}}, 0}$, and a set $\{\phi _i\}_{i=1,2, \dots , \ell _0}$ of normalized eigenfunctions of $L_{{\textbf{A}}, a}$, with corresponding eigenvalues $\mu _i^2, \nu _i^2 \in [-\frac{(d-2)^2}{4}, \eta _\ell +C_{{\textbf{A}}}-\frac{1}{2}]$ respectively, such that $\{e_i\}_{i=1,2, \dots , \ell _0}\cup \{e_{jk}\}_{j\ge \ell , k}$ and $\{\phi _i\}_{i=1,2, \dots , \ell _0}\cup \{\phi _{jk}\}_{j\ge \ell , k}$ are both bases of $L^2({\mathbb {S}}^{d-1})$.

In the above, we allow $\mu _i^2,\nu _i^2<0$ for $i<\ell _0$ for notational convenience.

#### Remark 1.2

Assumption 1.1 (1) is describing the typical asymptotic spectral picture after the occurrence of the Stark–Zeeman effect. First we notice a shift of the $\eta _j^2$’s. This is due to the presence of ${\textbf{A}}$ and *a*, but asymptotically the dominant order of the shift is given by ${\textbf{A}}$ (see e.g. [18, Lemma 2.1]), and this is the reason why $C_{\textbf{A}}$ does not depend on *a*. Then the free eigenvalues split in a cluster of new eigenvalues, and the total multiplicity remains equal to $m_j$. This last fact is generic in dimension 2, and it’s known to occur under some suitable symmetry assumptions in higher dimensions. Among the examples of potentials satisfying (1), we list:

- for any $d\ge 2$, the inverse-square potential, namely $L_{{\textbf{A}},a}$, with *a* constant;
- in $d=2$, provided ${\textbf{A}}\in W^{1, \infty }({\mathbb {S}}^{1},{\mathbb {R}}^2)$ and $a\in W^{1, \infty }({\mathbb {S}}^{1},{\mathbb {R}})$, see Sect. 5 for details;
- in $d\ge 3$, provided ${\textbf{A}}=0$ and *a* enjoys *zonal* symmetry [27, 40].

#### Remark 1.3

Assumption 1.1 (2) is about the low frequency part of the spectra: under this assumption, we can always complete the above families of eigenfunctions to orthonormal bases of $L^2({\mathbb {S}}^{d-1})$, by adding *exactly* the same number of low frequencies $j\le \ell _0$ in the two cases $L_{{\textbf{A}}, 0}$ and $L_{{\textbf{A}}, a}$. This is crucial in order to be able to give the definition of intertwining operators in Sect. 1.4 below. Although (2) could seem to be easily satisfied, we did not find in the literature any result along these lines. Hence we needed to perform a suitable analysis in order to give examples of potentials which fully satisfy Assumption 1.1, see Sect. 5.

From now on, we denote the index sets by

$$\begin{aligned} {\mathcal {I}}_\ell :=\{ (j,k): j\ge \ell , \ 1\le k \le m_j\}, \quad {\mathcal {I}}_{\ell }^{\perp }:=\{ 1,\dots ,\ell _0\}, \end{aligned}$$

so that $\{ e_\alpha \}_{\alpha \in {\mathcal {I}}_{\ell }^{\perp }\cup {\mathcal {I}}_\ell }$ and $\{ \phi _\alpha \}_{\alpha \in {{\mathcal {I}}_{\ell }^{\perp }} \cup {\mathcal {I}}_\ell }$ are two bases of $L^2({\mathbb {S}}^{d-1})$; we will also write $\sum _\alpha$ instead of $\sum _{\alpha \in {\mathcal {I}}_{\ell }^{\perp }\cup {\mathcal {I}}_\ell }$ and $\sum _{j,k}$ to mean $\sum _{j=\ell }^\infty \sum _{k=1}^{m_j}$ when the intent is clear.

We now aim to introduce the spectral measures associated to ${\mathcal {L}}_{{\textbf{A}},0}$ and ${\mathcal {L}}_{{\textbf{A}},a}$. We first notice that under Assumption 1.1, we have the following two orthogonal decompositions of $L^2({\mathbb {S}}^{d-1})$:

$$\begin{aligned} L^2({\mathbb {S}}^{d-1}) = \bigoplus _{{\alpha \in {\mathcal {I}}_{\ell }^{\perp }\cup {\mathcal {I}}_\ell }} \mathop {\textrm{span}}\limits \{ e_\alpha \} = \bigoplus _{{\alpha \in {\mathcal {I}}_{\ell }^{\perp }\cup {\mathcal {I}}_\ell }} \mathop {\textrm{span}}\limits \{ \phi _\alpha \}. \end{aligned}$$

Setting

$$\begin{aligned} {\mathcal {H}}_\alpha= &   \{ f(r) e_\alpha (\theta ) \mid f \in L^2({\mathbb {R}}_+; r^{d-1} \,\,\textrm{d}r) \}, \quad \alpha \in {\mathcal {I}}_{\ell }^{\perp }\cup {\mathcal {I}}_\ell , \\ \widetilde{{\mathcal {H}}}_\alpha= &   \{ f(r) \phi _\alpha (\theta ) \mid f \in L^2({\mathbb {R}}_+; r^{d-1} \,\,\textrm{d}r) \}, \quad \alpha \in {\mathcal {I}}_{\ell }^{\perp }\cup {\mathcal {I}}_\ell , \end{aligned}$$

we get that $L^2({\mathbb {R}}^d) = \bigoplus _{\alpha \in {\mathcal {I}}_{\ell }^{\perp }\cup {\mathcal {I}}_\ell } {\mathcal {H}}_\alpha = \bigoplus _{\alpha \in {\mathcal {I}}_{\ell }^{\perp }\cup {\mathcal {I}}_\ell } \widetilde{{\mathcal {H}}}_\alpha$. In other words, any function $f\in L^2({\mathbb {R}}^d)$ can be written as

$$\begin{aligned} f(x) = \sum _{{\alpha \in {\mathcal {I}}_{\ell }^{\perp }\cup {\mathcal {I}}_\ell }} f_\alpha (r) e_\alpha (\theta ) = \sum _{{\alpha \in {\mathcal {I}}_{\ell }^{\perp }\cup {\mathcal {I}}_\ell }} {\widetilde{f}}_\alpha (r) \phi _\alpha (\theta ) \end{aligned}$$

where $$f_\alpha (r) = \int _{{\mathbb {S}}^{d-1}} f(r,\theta ) {\bar{e}}_\alpha (\theta ) {\,\,\textrm{d}{\theta }}$$, and $${\widetilde{f}}_\alpha (r) = \int _{{\mathbb {S}}^{d-1}} f(r,\theta ) {\bar{\phi }}_\alpha (\theta ) {\,\,\textrm{d}{\theta }}$$.

We now define the following projection operators to each subspace:

$$\begin{aligned} \begin{aligned} {\mathbb {H}}_{\alpha }&:={\mathcal {L}}_{{\textbf{A}},0}|_{{{\mathcal {H}}}_{\alpha }}=-\partial _r^2-\frac{d-1}{r}\partial _r+\frac{\mu _{\alpha }^2}{r^2}, \quad \alpha \in {\mathcal {I}}_{\ell }^{\perp }\cup {\mathcal {I}}_\ell , \\ \widetilde{\mathbb {H}}_{\alpha }&:={\mathcal {L}}_{{\textbf{A}},a}|_{\widetilde{{\mathcal {H}}}_{\alpha }}=-\partial _r^2-\frac{d-1}{r}\partial _r+\frac{\nu _{\alpha }^2}{r^2}, \quad \alpha \in {\mathcal {I}}_{\ell }^{\perp }\cup {\mathcal {I}}_\ell ,. \end{aligned} \end{aligned}$$

It is well-known that ${\mathbb {H}}_{\alpha }$ and $\widetilde{{\mathbb {H}}}_{\alpha }$ formally have eigenfunctions

$$\begin{aligned} E_{\alpha }(r, \lambda )=(r\lambda )^{-\frac{d-2}{2}} J_{\widetilde{\mu }_{\alpha }}(\lambda r); \quad {\widetilde{E}}_{\alpha }(r, \lambda )=( r\lambda )^{-\frac{d-2}{2}} J_{\widetilde{\nu }_{\alpha }}(\lambda r), \end{aligned}$$

where the $J_{\nu }$ are Bessel functions of the first kind of order $\nu$, and

$$\begin{aligned}\widetilde{\mu }_{\alpha }=\sqrt{\mu _{\alpha }^{2}+\Big (\frac{d-2}{2}\Big )^{2}}, \quad \widetilde{\nu }_{\alpha }=\sqrt{\nu _{\alpha }^{2}+\Big (\frac{d-2}{2}\Big )^{2}}.\end{aligned}$$

Their corresponding eigenvalue is $\lambda ^2$, i.e.

$$\begin{aligned} {\mathbb {H}}_{\alpha } E_{\alpha }(r, \lambda )=\lambda ^2 E_{\alpha }(r, \lambda ), \quad \widetilde{\mathbb {H}}_{\alpha }{\widetilde{E}}_{\alpha }(r, \lambda )=\lambda ^2 {\widetilde{E}}_{\alpha }(r, \lambda ). \end{aligned}$$

For a radial function $f(r) \in L^2({\mathbb {R}}^+,r^{d-1}\,\,\textrm{d}r)$, we then define its Hankel transform of order $\nu \ge 0$ as

1.3 $$\begin{aligned} {\mathscr {H}}_{\nu }f(\lambda )=\int _0^\infty (r\lambda )^{-\frac{d-2}{2}} J_{\nu }(r\lambda ) f(r) r^{d-1}\,\,\textrm{d}r. \end{aligned}$$

It is easy to see that

$$\begin{aligned} {\mathscr {H}}_{\nu }{\mathscr {H}}_{\nu }=I, \qquad \Vert f\Vert _{L^2({\mathbb {R}}^+,r^{d-1}\,\,\textrm{d}r)}=\Vert {\mathscr {H}}_{\nu }f\Vert _{L^2({\mathbb {R}}^+, \lambda ^{d-1}\,\,\textrm{d}\lambda )}. \end{aligned}$$

Furthermore, Hankel transforms diagonalize the differential operators ${\mathbb {H}}_{\alpha }$ and $\widetilde{\mathbb {H}}_{\alpha }$:

$$\begin{aligned} {\mathscr {H}}_{\widetilde{\mu }_{\alpha }}({\mathbb {H}}_{\alpha } f)(\lambda ) =\lambda ^2 {\mathscr {H}}_{\widetilde{\mu }_{\alpha }} f(\lambda ), \quad {\mathscr {H}}_{\widetilde{\nu }_{\alpha }}(\widetilde{\mathbb {H}}_{\alpha } f)(\lambda ) =\lambda ^2 {\mathscr {H}}_{\widetilde{\nu }_{\alpha }} f(\lambda ). \end{aligned}$$

Therefore, the spectral measures associated to ${\mathcal {L}}_{{\textbf{A}},a}$ and ${\mathcal {L}}_{{\textbf{A}},0}$ are given by

$$\begin{aligned} \,\,\textrm{d}E_{\lambda }({\textbf{A}}, 0) f(r, \theta )&= \sum _{{\alpha \in {\mathcal {I}}_{\ell }^{\perp }\cup {\mathcal {I}}_\ell }} {\mathscr {H}}_{\widetilde{\mu }_{\alpha }} f_{\alpha }(\lambda ) e_{\alpha }(\theta ),\quad \text {and} \\ \,\,\textrm{d}E_{\lambda }({\textbf{A}}, a) f(r, \theta )&= \sum _{{\alpha \in {\mathcal {I}}_{\ell }^{\perp }\cup {\mathcal {I}}_\ell }} {\mathscr {H}}_{\widetilde{\nu }_{\alpha }} {\widetilde{f}}_{\alpha }(\lambda ) \phi _{\alpha }(\theta ). \end{aligned}$$

### Intertwining Operators

We are now ready to introduce the core of this manuscript. We start by defining the following families of linear angular operators:

1.4 $$\begin{aligned} W^\theta _{\alpha }:\mathop {\textrm{span}}\limits \{e_{\alpha }(\theta )\}&\rightarrow \mathop {\textrm{span}}\limits \{\phi _{\alpha }(\theta )\} \nonumber \\ W_{\alpha }^\theta (e_{\alpha }(\theta ))&= \phi _{\alpha }(\theta ). \end{aligned}$$

The dual operators $\{W_{\alpha }^{\theta ,*}\}_{\alpha \in {\mathcal {I}}_{\ell }^{\perp }\cup {\mathcal {I}}_\ell }$ are given by

$$\begin{aligned} W^{\theta ,*}_{\alpha }:\mathop {\textrm{span}}\limits \{\phi _{\alpha }(\theta )\}&\rightarrow \mathop {\textrm{span}}\limits \{e_{\alpha }(\theta )\} \nonumber \\ W_{\alpha }^{\theta , *}(\phi _{\alpha }(\theta ))&= e_{\alpha }(\theta ). \end{aligned}$$

#### Remark 1.4

Notice that the above operators in are not uniquely defined: indeed, definition (1.4) depends on the way we order the eigenfunctions $e_\alpha ,\varphi _\alpha$, when the corresponding eigenvalues are repeated (see Assumption 1.1 above). Nevertheless, we are able to prove that any of these choices of the order leads to the definition of intertwining operators with suitable boundedness properties in $L^p$.

For each fixed $\alpha \in {\mathcal {I}}_{\ell }^{\perp }\cup {\mathcal {I}}_\ell$, $W_{\alpha }^\theta$ and $W_{\alpha }^{\theta ,*}$ are unitary operators on $L^2({\mathbb {S}}^{d-1})$ since both $e_{\alpha }(\theta )$ and $\phi _{\alpha }(\theta )$ are normalized.

Next, we define a family of radial operators $\{W_{\alpha }^r\}_{\alpha \in {\mathcal {I}}_{\ell }^{\perp }\cup {\mathcal {I}}_\ell }$ on $L^2(r^{d-1}\,\,\textrm{d}r)$ as follows:

1.5 $$\begin{aligned} W_{\alpha }^r:={\mathscr {H}}_{{\widetilde{\nu }_{\alpha }}}{\mathscr {H}}_{\widetilde{\mu }_{\alpha }}. \end{aligned}$$

As for the dual operators $W^{r,*}_\alpha$, those are given by

$$\begin{aligned} W_{\alpha }^{r,*}={\mathscr {H}}_{\widetilde{\mu }_{\alpha }}{\mathscr {H}}_{\widetilde{\nu }_{\alpha }}. \end{aligned}$$

Thanks to the properties of the Hankel transform, we see that for each fixed *j*, $W_{\alpha }^r$ and $W_{\alpha }^{r, *}$ are unitary operators on $L^2(r^{d-1} \,\,\textrm{d}r)$.

We can finally give the main definition of this manuscript.

#### Definition 1.5

*(Intertwining operators).* Given $f \in L^2({\mathbb {R}}^d)$, with expansions

$$\begin{aligned} f(x) = \sum _{{\alpha \in {\mathcal {I}}_{\ell }^{\perp }\cup {\mathcal {I}}_\ell }} f_\alpha (r) e_\alpha (\theta ) = \ \sum _{{\alpha \in {\mathcal {I}}_{\ell }^{\perp }\cup {\mathcal {I}}_\ell }} {\widetilde{f}}_\alpha (r) \phi _\alpha (\theta ), \end{aligned}$$

we define the intertwining operators *W*, $W^*$ on $L^2({\mathbb {R}}^d)$ by

1.6 $$\begin{aligned} Wf(x)&:=\sum _{{\alpha \in {\mathcal {I}}_{\ell }^{\perp }\cup {\mathcal {I}}_\ell }} W^r_\alpha f_\alpha (r) W^\theta _\alpha (e_\alpha (\theta )) = \sum _{{\alpha \in {\mathcal {I}}_{\ell }^{\perp }\cup {\mathcal {I}}_\ell }} {\mathscr {H}}_{{\widetilde{\nu }_{\alpha }}}{\mathscr {H}}_{\widetilde{\mu }_{\alpha }}f_\alpha (r) \phi _\alpha (\theta )\, \end{aligned}$$

with its dual operator $W^*$ formally given by

1.7 $$\begin{aligned} W^*f(x)&:=\sum \limits _{{\alpha \in {\mathcal {I}}_{\ell }^{\perp }\cup {\mathcal {I}}_\ell }} W^{r,*}_\alpha {\widetilde{f}}_\alpha (r) W^{\theta ,*}_\alpha (\phi _\alpha (\theta ))= \sum \limits _{{\alpha \in {\mathcal {I}}_{\ell }^{\perp }\cup {\mathcal {I}}_\ell }} {\mathscr {H}}_{{\widetilde{\mu }_{\alpha }}}{\mathscr {H}}_{\widetilde{\nu }_{\alpha }}f_\alpha (r) e_\alpha (\theta ).\nonumber \\ \end{aligned}$$

In order to check the duality, notice that, for any $f, g \in L^2({\mathbb {R}}^d)$, one has

$$\begin{aligned} \langle Wf, g\rangle&=\sum _{\alpha }\langle {\mathscr {H}}_{\widetilde{\nu }_\alpha }{\mathscr {H}}_{\widetilde{\mu }_\alpha } f_\alpha , {\widetilde{g}}_\alpha \rangle _{\widetilde{\mathcal {H}}_\alpha }=\sum _{\alpha }\langle f_\alpha , {\mathscr {H}}_{\widetilde{\mu }_\alpha }{\mathscr {H}}_{\widetilde{\nu }_\alpha }{\widetilde{g}}_\alpha \rangle _{{\mathcal {H}}_\alpha }= \langle f, W^*g\rangle . \end{aligned}$$

As the Hankel transform is unitary, and both $\{e_\alpha \}_{\alpha \in {\mathcal {I}}_{\ell }^{\perp }\cup {\mathcal {I}}_\ell }$ and $\{\phi _\alpha \}_{\alpha \in {\mathcal {I}}_{\ell }^{\perp }\cup {\mathcal {I}}_\ell }$ are bases, it is easy to see that *W* is also unitary, i.e.

1.8 $$\begin{aligned} W W^*= W^*W =I. \end{aligned}$$

We now show that the above operator enjoy the above mentioned intertwining property.

#### Lemma 1.6

(Intertwining property). The operator *W* defined in (1.6) satisfies the following intertwining property in $L^2({\mathbb {R}}^d)$: for any bounded Borel-measurable function *F*,

1.9 $$\begin{aligned} F({\mathcal {L}}_{{\textbf{A}},a})=WF({\mathcal {L}}_{{\textbf{A}},0})W^*. \end{aligned}$$

#### Proof

By (1.8), it is sufficient to show that $F({\mathcal {L}}_{{\textbf{A}},a}) W=WF({\mathcal {L}}_{{\textbf{A}},0})$. By the definition, we have for the left-hand side

$$\begin{aligned} F({\mathcal {L}}_{{\textbf{A}},a})W f(r,\theta )&=F\big ({\mathcal {L}}_{{\textbf{A}},a}\big ) \Big (\sum _\alpha {\mathscr {H}}_{{\widetilde{\nu }_{\alpha }}}{\mathscr {H}}_{\widetilde{\mu }_{\alpha }} f_{\alpha }(r ) \phi _{\alpha }(\theta )\Big ) \\&=\sum _\alpha F(\widetilde{\mathbb {H}}_{\alpha }) {\mathscr {H}}_{{\widetilde{\nu }_{\alpha }}}{\mathscr {H}}_{\widetilde{\mu }_{\alpha }} f_{\alpha }(r ) \phi _{\alpha }(\theta ) \\&=\sum _\alpha {\mathscr {H}}_{\widetilde{\nu }_{\alpha }}{\mathscr {H}}_{\widetilde{\nu }_{\alpha }}F({\mathbb {H}}_{\alpha } ){\mathscr {H}}_{\widetilde{\nu }_{\alpha }}{\mathscr {H}}_{\widetilde{\mu }_{\alpha }} f_{\alpha }(r ) \phi _{\alpha }(\theta )) \\&=\sum _\alpha {\mathscr {H}}_{{\widetilde{\nu }_{\alpha }}}\left[ F(\lambda ^2) ({\mathscr {H}}_{\widetilde{\mu }_{\alpha }}f_{\alpha })(\lambda )\right] (r ) \phi _{\alpha }(\theta ), \end{aligned}$$

while the right-hand side is

$$\begin{aligned} WF({\mathcal {L}}_{{\textbf{A}},0})f(r,\theta )&=W \bigg (\sum _\alpha F({\mathbb {H}}_{\alpha }) f_{\alpha }(r )e_{\alpha }(\theta )\bigg ) \\&=\sum _\alpha {\mathscr {H}}_{\widetilde{\nu }_{\alpha }}{\mathscr {H}}_{\widetilde{\mu }_{\alpha }}\left[ F({\mathbb {H}}_{\alpha }) f_{\alpha }(\cdot )\right] (r )\phi _{\alpha }(\theta ) \\&=\sum _\alpha {\mathscr {H}}_{{\widetilde{\nu }_{\alpha }}}\left[ F(\lambda ^2) \left( {\mathscr {H}}_{\widetilde{\mu }_{\alpha }}f_{\alpha }\right) (\lambda )\right] (r )\phi _{\alpha }(\theta ). \end{aligned}$$

The proof is hence complete. $\square$

## Main Results

We showed in the previous section that the intertwining operators defined in (1.6) and (1.7) are bounded in $L^2({\mathbb {R}}^d)$. However, to get the $L^p$-boundedness of those operators for $p\ne 2$, we need stronger spectral conditions than those in Assumption 1.1 on the spherical operators $L_{{\textbf{A}},0}$ and $L_{{\textbf{A}},a}$. Roughly speaking, we need to control the asymptotic size of the separation between the eigenvalues $\mu _{jk}^2, \nu _{jk}^2$. Notice that ${\textbf{A}}$ is fixed in the two cases; in fact, we believe that the intertwining operators between ${\mathcal {L}}_{{\textbf{A}}_1,0}$ and ${\mathcal {L}}_{{\textbf{A}}_2,0}$ are not bounded in $L^p$ for any nontrivial ${\textbf{A}}_1,{\textbf{A}}_2$, as their eigenvalues $\mu _{jk}^2, \nu _{jk}^2$ (appropriately paired up) will not be close enough to each other as *j* goes to infinity (see the details in the sequel).

### Discrete Mulitiplier Bases

In order to state our main results, we start by some preliminary notations and definitions. For any doubly indexed sequence $\{{\mathcal {C}}_{\alpha }\}_{\alpha \in {\mathcal {I}}_\ell }$, we define its average sequence $\{{\overline{{\mathcal {C}}}}_j\}_{j\ge \ell }$ by

$$\begin{aligned} {\overline{{\mathcal {C}}}}_j=\frac{1}{m_j} \sum _{k=1}^{m_j} {\mathcal {C}}_{jk} \end{aligned}$$

Then we define the forward finite difference operator with respect to *j* acting on sequences $\{{\overline{{\mathcal {C}}}}_{j}\}_{j \ge \ell }$ by

$$\begin{aligned} {\mathfrak {D}}{\overline{{\mathcal {C}}}}_{j}:={\overline{{\mathcal {C}}}}_{j+1} - {\overline{{\mathcal {C}}}}_{j}, \quad {\mathfrak {D}}^N {\overline{{\mathcal {C}}}}_{j}:={\mathfrak {D}}^{N-1} ({\mathfrak {D}}{\overline{{\mathcal {C}}}}_{j}). \end{aligned}$$

#### Definition 2.1

We call a basis $\{\psi _{\alpha }\}_{\alpha \in {\mathcal {I}}_{\ell }^{\perp }\cup {\mathcal {I}}_\ell }$ on $L^2({\mathbb {S}}^{d-1})$ a *discrete multiplier basis* and write $\{\psi _{\alpha }\}_{\alpha \in {\mathcal {I}}_{\ell }^{\perp }\cup {\mathcal {I}}_\ell } \in \mathcal {DMB}$ if whenever $\{{\mathcal {C}}_{\alpha }\}_{\alpha \in {\mathcal {I}}_\ell }$ is a sequence of complex numbers satisfying

2.1 $$\begin{aligned} \sup _{j\ge \ell } \left| {\overline{{\mathcal {C}}}}_{j}\right|&\lesssim 1, \end{aligned}$$

2.2 $$\begin{aligned} \sup _{L\in {\mathbb {N}}:2^L \ge \ell } 2^{(N-1)L} \sum _{j=2^L}^{2 ^{L+1}}\left| {\mathfrak {D}}^N {\overline{{\mathcal {C}}}}_{j}\right|&\lesssim 1,\quad N=1,2, \dots , \left\lfloor \frac{d-1}{2}\right\rfloor , \end{aligned}$$

we have for all $1<p<\infty$ the following inequality,

$$\begin{aligned} \bigg \Vert \sum _{j=\ell }^\infty \sum _{k=1}^{m_j}{\mathcal {C}}_{jk} F_{jk} \psi _{jk}(\theta )\bigg \Vert _{L^{p}({\mathbb {S}}^{d-1})} \lesssim \bigg \Vert \sum _{j=\ell }^\infty \sum _{k=1}^{m_j} F_{jk} \psi _{jk}(\theta )\bigg \Vert _{L^{p}({\mathbb {S}}^{d-1})}. \end{aligned}$$

#### Remark 2.2

Let $\{\psi _\alpha \}_\alpha$ be the eigenfunctions of $L_{{\textbf{A}},a}$. When ${{\textbf{A}}}=0$ and $a(\theta )=0$, $\{\psi _{\alpha }\}$ are the spherical harmonics on ${\mathbb {S}}^{d-1}$, and $\{\psi _{\alpha }\}_{\alpha }\in \mathcal {DMB}$ by the results in [4]. More generally, for any ${\textbf{A}}\in C^{\infty }({\mathbb {S}}^{d-1}; {\mathbb {R}}^{d})$ and $a \in C^{\infty }({\mathbb {S}}^{d-1}; {\mathbb {R}})$, $\{\psi _{\alpha }\}_\alpha \in \mathcal {DMB}$ by [38, Theorem 5.3.1].

We now state a new fundamental tool for our main results.

#### Lemma 2.3

(Variable coefficients discrete multiplier theorem). Let $\{{\mathcal {C}}_{\alpha }(\theta )\}_{\alpha \in {\mathcal {I}}_\ell }$ be a sequence of bounded functions belonging to $C^{d-1}({\mathbb {S}}^{d-1})$ and satisfying the following conditions:

$$\begin{aligned} \sup _{j, \theta }|{\overline{{\mathcal {C}}}}_{j}(\theta )|<C, \quad \sup _{j, \theta }\left| \partial _\theta ^{d-1}{\overline{{\mathcal {C}}}}_{j}(\theta )\right|<C, \\ \sup _{\theta , 2^L \ge \ell } 2^{(N-1)L} \sum _{j=2^L }^{2 ^{L+1}}\left| {\mathfrak {D}}^N {\overline{{\mathcal {C}}}}_{j}(\theta )\right|< C, \\ \sup _{\theta , 2^L \ge \ell } 2^{(N-1)L} \sum _{j=2^L}^{2 ^{L+1}}\left| {\mathfrak {D}}^N \partial _\theta ^{d-1} {\overline{{\mathcal {C}}}}_{j}(\theta )\right| <C, \end{aligned}$$

for $N=1,2, \dots , \left\lfloor \frac{d-1}{2} \right\rfloor$. If $\{\psi _{\alpha }\} \in \mathcal {DMB}$, then for every $1<p<\infty$,

$$\begin{aligned} \bigg \Vert \sum _{j = \ell }^\infty \sum _{k=1}^{m_j} {\mathcal {C}}_{jk}(\theta ) F_{jk} \psi _{jk}(\theta )\bigg \Vert _{L^{p}({\mathbb {S}}^{d-1})} \lesssim \bigg \Vert \sum _{j = \ell }^\infty \sum _{k=1}^{m_j} F_{jk} \psi _{jk}(\theta )\bigg \Vert _{L^{p}({\mathbb {S}}^{d-1})}. \end{aligned}$$

#### Proof

Let $\theta =\theta (\theta _1,\dots ,\theta _{d-1})\in {\mathbb {S}}^{d-1}$ be the standard parameterization of the sphere, and abusively write ${\mathcal {C}}_{jk}(\theta _1,\dots ,\theta _{d-1}):= {\mathcal {C}}_{jk}(\theta (\theta _1,\dots ,\theta _{d-1}))$. By the Fundamental Theorem of Calculus, we can write

$$\begin{aligned} {\mathcal {C}}_{jk}(\theta )&= {\mathcal {C}}_{jk}(0,\theta _2,\dots ,\theta _{d-1}) +\int _0^{\theta _1} (\partial _\theta {\mathcal {C}}_{jk}\partial _{\theta _1} \theta )(\theta _1',\theta _2,\dots ,\theta _{d-1}){\,\,\textrm{d}{\theta }}_{1}' \end{aligned}$$

We can repeat this again for the first term ${\mathcal {C}}_{jk}(0,\theta _2,\dots ,\theta _{d-1})$ to get

$$\begin{aligned} {\mathcal {C}}_{jk}(0,0,\theta _3,\dots ,\theta _{d-1}) +\int _0^{\theta _2} \partial _\theta {\mathcal {C}}_{jk}(0,\theta _2',\theta _3,\dots ,\theta _{d-1})\partial _{\theta _2} \theta (0,\theta _2',\theta _3,\dots ,\theta _{d-1}){\,\,\textrm{d}{\theta }}_{2}', \end{aligned}$$

and for the integral term $\int _0^{\theta _1} (\partial _\theta {\mathcal {C}}_{jk}\partial _{\theta _1} \theta )(\theta _1',\theta _2,\dots ,\theta _{d-1}){\,\,\textrm{d}{\theta }}_{1}'$ to get

$$\begin{aligned}  &   \int _0^{\theta _1} (\partial _\theta {\mathcal {C}}_{jk}\partial _{\theta _1} \theta )(\theta _1',0,\theta _3,\dots ,\theta _{d-1}){\,\,\textrm{d}{\theta }}_{1}' \\  &   \quad + \int _0^{\theta _2}\int _0^{\theta _1} (\partial _\theta ^2 {\mathcal {C}}_{jk} \partial _{\theta _1} \theta \partial _{\theta _2}\theta + \partial _\theta {\mathcal {C}}_{jk} \partial _{\theta _1}\partial _{\theta _2}\theta )(\theta _1',\theta _2',\theta _3,\dots ,\theta _{d-1}){\,\,\textrm{d}{\theta }}_{1}'\,\,\textrm{d}\theta _2'. \end{aligned}$$

We can inductively repeat this until we obtain terms of the form

$$\begin{aligned} c\int _0^{\theta _{i_1}}\dots \int _0^{\theta _{i_N}} G(\theta '_{i_1} ,\dots ,\theta '_{i_N}) \,\,\textrm{d}\theta '_{i_1} \dots \,\,\textrm{d}\theta '_{i_N} \end{aligned}$$

where *c* is a combinatorial constant, $0\le M\le N\le d-1$, and $G(\theta '_{i_1},\dots ,\theta '_{i_N})$ is the product of $\partial ^{M}_\theta {\mathcal {C}}_{jk}$ and various derivatives of the parameterisation $\theta$, all evaluated at the point

$$\begin{aligned} (0,\dots ,0,\theta '_{i_1},0,\dots ,0,\theta _{i_N}',0,\dots ,0)\in {\mathbb {R}}^{d-1}, \end{aligned}$$

i.e. the point whose $i_k^\text {th}$ component is equal to $\theta _{i_k}'$, and all other components equal to 0. A precise enumeration could be given with the Faà di Bruno formula, but the important point is that every term depends on the coordinates $\theta _1,\dots ,\theta _{d-1}$ only in the integration limits. Thus, every integrand $G(\theta '_{i_1},\dots ,\theta '_{i_N})$ can be treated as a constant coefficient multiplier, as they satisfy (2.1) and (2.2) for each fixed choice of $\theta '_{i_1},\dots ,\theta '_{i_N}$. Hence, by applying Definition 2.1, we have that for any $1<p<\infty$,

$$\begin{aligned}&\Big \Vert \sum _{j,k} {\mathcal {C}}_{jk}(\theta )F_{jk} \psi _{jk}(\theta )\Big \Vert _{L^p} \\&\quad \lesssim \Big \Vert \sum _{j,k}{\mathcal {C}}_{jk}(0,\dots ,0) F_{jk} \psi _{jk}(\theta )\Big \Vert _{L^p} \\&\qquad +\!\!\sum _{M,N,i_1,\dots ,i_N}\int _0^{\pi }\!\cdots \! \int _0^{\pi }\!\int _0^{2\pi } \Big \Vert \sum _{j,k}G(\theta '_{i_1} ,\dots ,\theta '_{i_N}) F_{jk} \psi _{jk}(\theta ) \Big \Vert _{L^p} \,\,\textrm{d}\theta '_{i_1} \dots \,\,\textrm{d}\theta '_{i_N} \\&\quad \lesssim \bigg \Vert \sum _{j,k} F_{jk} \psi _{jk}(\theta )\bigg \Vert _{L^{p}},\end{aligned}$$

which was what we wanted. $\square$

### Proper Perturbations

#### Definition 2.4

*(Proper perturbation).*Assume that $L_{{\textbf{A}}, a}$ and $L_{{\textbf{A}}, 0}$ satisfy Assumption 1.1. We call $L_{{\textbf{A}}, a}$ is a *proper perturbation* of $L_{{\textbf{A}},0}$ if

1. There exists a constant $C_a$ that depends only on ${\textbf{A}}$ and *a* such that for all $j\ge \ell$,
  $$\begin{aligned} \left| \nu _{jk}^2 - \mu _{jk}^2-C_a\right| \le \frac{C}{j} \end{aligned}$$
  for all $1 \le k \le m_j$, where *C* is a constant independent of *j* and *k*.
2. The corresponding eigenfunctions $\phi _{jk}$ and $e_{jk}$ $j\ge \ell$ can be written
  $$\begin{aligned} \phi _{jk}(\theta )=e_{jk}(\theta )(1+ R_{jk}(\theta )),\end{aligned}$$
  where $R_{jk}$ satisfies with $N=\big \lfloor \frac{d-1}{2}\big \rfloor +1$ the following estimates:
  - $$\sup _{\theta }|{\overline{R}}_{j}(\theta )|=O(\frac{1}{j})$$ as $$j\rightarrow \infty $$, where $${\overline{R}}_j=\frac{1}{m_j} \sum _{k=1}^{m_j} R_{jk}$$,
  - $$\sup _{2^L\ge \ell , \theta }2^{(N-1)L}\sum _{j=2^L}^{2^{L+1}}|{\mathfrak {D}}^N {\overline{R}}_{j}(\theta )| \lesssim \ell ^{-1}$$,
  - $$\sup _{2^L \ge \ell , \theta \in {\mathbb {S}}^{d-1}}|\partial _\theta ^{d-1} {\overline{R}}_{j}(\theta )|\lesssim \ell ^{-1}$$,
  - $$\sup _{2^L\ge \ell , \theta }2^{(N-1)L}\sum _{j=2^L}^{2^{L+1}}|{\mathfrak {D}}^N \partial _\theta ^{d-1} {{\overline{R}}}_{j}(\theta )| \lesssim \ell ^{-1}$$.

#### Remark 2.5

In some cases, in order to estimate the derivatives of the reminder terms, we can further decompose $R_{jk}=\sum _{m=1}^d R_{jk}^{m}$ with the $m^{\text {th}}$ reminder term $R_{jk}^m$ satisfying

$$\begin{aligned} \Vert R_{jk}^{m}\Vert _{L^\infty } =O\Big (\frac{1}{j^m}\Big ). \end{aligned}$$

For $m\le d-1$, we can control the derivatives using the explicit formula. For $R_{jk}^d$, we can use triangle inequality and the fact that $\sum _{jk} \frac{1}{j^d}\lesssim \sum _{j}\frac{1}{j^2}<\infty$ to get the boundedness in $L^p$. We will see this in Sect. 5.2.

#### Remark 2.6

When $d=2$, ${\textbf{A}}\in W^{1, \infty }({\mathbb {S}}^1)$ and $a\in W^{1, \infty }({\mathbb {S}}^1)$, $L_{{\textbf{A}}, a}$ is a proper perturbation of $L_{{\textbf{A}}, 0}$ by the results in [18].

#### Remark 2.7

An important question that we will not address in the present paper is the following: for a fixed ${\textbf{A}}$, what is the minimal regularity of $a(\theta )$ such that $L_{{\textbf{A}}, a}$ is a proper perturbation of $L_{{\textbf{A}},0}$?

#### Lemma 2.8

Assume that $\{e_\alpha \} \in \mathcal {DMB}$. If $L_{{\textbf{A}}, a}$ is a proper perturbation of $L_{{\textbf{A}}, 0}$, then $\{\phi _\alpha \} \in \mathcal {DMB}$. That is, whenever $\{{\mathcal {C}}_{jk}\}_{(j,k) \in {\mathcal {I}}_\ell }$ satisfies the estimates (2.1) and (2.2), the perturbed eigenfunctions $\{\phi _{jk}\}$ satisfy

$$\begin{aligned} \bigg \Vert \sum _{j = \ell }^\infty \sum _{k=1}^{m_j}{\mathcal {C}}_{jk} F_{jk} \phi _{jk}(\theta )\bigg \Vert _{L^{p}({\mathbb {S}}^{d-1})} \lesssim \bigg \Vert \sum _{j = \ell }^\infty \sum _{k=1}^{m_j} F_{jk} \phi _{jk}(\theta )\bigg \Vert _{L^{p}({\mathbb {S}}^{d-1})}. \end{aligned}$$

#### Proof

From $\phi _{jk}=e_{jk}(\theta )(1 + R_{jk}(\theta ))$, triangle inequality and $\{e_\alpha \}_\alpha \in \mathcal {DMB}$, we apply Lemma 2.3 with ${\mathcal {C}}_{jk} R_{jk}$ as the variable coefficients to get

$$\begin{aligned} \bigg \Vert \sum _{j,k} {\mathcal {C}}_{jk} F_{jk} \phi _{jk}\bigg \Vert _{L^p} \lesssim \bigg \Vert \sum _{j,k} {\mathcal {C}}_{jk} F_{jk} e_{jk} \bigg \Vert _{L^p} + \bigg \Vert \sum _{j,k} {\mathcal {C}}_{jk} R_{jk} F_{jk} e_{jk} \bigg \Vert _{L^p} \lesssim \bigg \Vert \sum _{j,k} F_{jk} e_{jk} \bigg \Vert _{L^p}. \end{aligned}$$

To finish, we write $e_{jk} = \phi _{jk} - e_{jk} R_{jk}$ and use the fact that $R_{jk}$ is small for $j\gg 1$,

$$\begin{aligned} \bigg \Vert \sum _{j,k} F_{jk} e_{jk}\bigg \Vert _{L^{p}}&\le \bigg \Vert \sum _{j,k} F_{jk} \phi _{jk}\bigg \Vert _{L^{p}}+\frac{C}{\ell }\bigg \Vert \sum _{j,k} F_{jk} e_{jk}\bigg \Vert _{L^{p}}, \end{aligned}$$

so the second term on the right hand side can be absorbed into the left side. So the result is proven.

$$\square $$

### Main Result

We are now ready to state our main result. From now on, we let $n(d):=\frac{d-2}{2}$.

#### Theorem 2.9

($L^p$-boundedness of *W* and $W^*$). Let $d\ge 2$, and $L_{{\textbf{A}}, 0}$, $L_{{\textbf{A}}, a}$ satisfy Assumption 1.1. Assume that the eigenfunctions of $L_{{\textbf{A}},0}$, $\{e_\alpha \}_\alpha \in \mathcal {DMB}$ and $L_{{{\textbf{A}}, a}}$ is a proper perturbation of $L_{{{\textbf{A}}, 0}}$. Then *W* defined in (1.6) is bounded on $L^p({\mathbb {R}}^d)$ for $p\in (1,\infty )$ satisfying

$$\begin{aligned} \frac{n(d)-\widetilde{\nu }_1}{d}<\frac{1}{p}<\frac{d-n(d)+\widetilde{\mu }_1}{d}, \end{aligned}$$

and $W^*$ defined in (1.7) is bounded on $L^p({\mathbb {R}}^d)$ for $p\in (1,\infty )$ satisfying

$$\begin{aligned} \frac{n(d)-\widetilde{\mu }_1}{d}<\frac{1}{p}<\frac{d-n(d)+\widetilde{\nu }_1}{d}, \end{aligned}$$

and $\widetilde{\mu }_1=\sqrt{\lambda _1({\textbf{A}},0)+n(d)^2}$, $\widetilde{\nu }_1=\sqrt{\lambda _1({\textbf{A}},a)+n(d)^2}$. In particular, if $\widetilde{\mu }_1, \widetilde{\nu }_1 \ge n(d)$, then *W* and $W^*$ are bounded on $L^p({\mathbb {R}}^d)$ for all $1<p<\infty$.

#### Remark 2.10

Note that the restriction on $p$ depends on the angular operators $L_{{\textbf{A}},0}, L_{{\textbf{A}},a}$ only through their first eigenvalues.

#### Remark 2.11

Owing to our use of the theory of singular integrals and the discrete multiplier theorem, we are unable to prove the endpoint cases $p=1,\infty$ with this method.

#### Remark 2.12

For $d\ge 3$, Theorem 2.9 includes the inverse square potential which is given by ${\textbf{A}}=0$ and *a* constant. For more general potentials, our result shows that to study ${\mathcal {L}}_{{\textbf{A}},a}$ it suffices to understand ${\mathcal {L}}_{{\textbf{A}},0}$ and the perturbation theory for the angular component.

### The Two-dimensional Case

In dimension $d=2$, the spectral assumptions in our main theorem are generically satisfied, which leads to the following quite complete result. Let us define $A,{\widetilde{a}}$, and ${\widetilde{A}}$ by

$$\begin{aligned} \begin{aligned} A(\theta )&={\textbf{A}}(\cos \theta ,\sin \theta )\cdot (-\sin \theta ,\cos \theta ),\\ {\widetilde{a}}&=\frac{1}{2\pi }\int _0^{2\pi } a(\theta ){\,\,\textrm{d}{\theta }}, \qquad {\widetilde{A}}=\frac{1}{2\pi }\int _0^{2\pi } A(\theta )\,\,\textrm{d}\theta . \end{aligned} \end{aligned}$$

#### Theorem 2.13

Let $a\in W^{1,\infty }({\mathbb {S}}^{1},{\mathbb {R}})$, ${\textbf{A}}\in W^{1,\infty }({\mathbb {S}}^{1},{\mathbb {R}}^2)$ satisfying (1.1) and (1.2) hold. If ${\widetilde{A}} \notin \frac{1}{2} {\mathbb {Z}}$ or ${\widetilde{A}} \in \frac{1}{2} {\mathbb {Z}}$ and $a(\theta )$ is symmetric across $\theta =\pi$ (i.e. $a(\pi -\theta )=a(\pi +\theta )$ for all $\theta \in [0,\pi ]$), *W* and $W^*$ defined in (1.6) and (1.7) are $L^p({\mathbb {R}}^2)$-bounded for all $1<p<\infty$.

Theorem 2.13 will be proven in Sect. 5 using Theorem 2.9. We now state some of the consequences of Theorem 2.13.

For the wave propagator, the authors in [21] has proved the following estimate for the electric-free operator,

$$\begin{aligned} \bigg \Vert \frac{\sin (t\sqrt{{\mathcal {L}}_{{\textbf{A}},0}})}{\sqrt{{\mathcal {L}}_{{\textbf{A}},0}}}\varphi (\sqrt{{\mathcal {L}}_{{\textbf{A}},0}})f\bigg \Vert _{L^\infty ({\mathbb {R}}^2)} \le C(1+|t|)^{-\frac{1}{2}}\Vert f\Vert _{L^1({\mathbb {R}}^2)}, \end{aligned}$$

where $\text {supp}\;\varphi \subset [1/2,2].$ Interpolating this with trivial $L^2$ bound, we have for any $1\le p\le 2$

$$\begin{aligned} \bigg \Vert \frac{\sin (t\sqrt{{\mathcal {L}}_{{\textbf{A}},0}})}{\sqrt{{\mathcal {L}}_{{\textbf{A}},0}}}\varphi \big (\sqrt{{\mathcal {L}}_{{\textbf{A}},0}}\big )f\bigg \Vert _{L^{p'}({\mathbb {R}}^2)} \le C(1+|t|)^{-\frac{1}{2}(\frac{1}{p}-\frac{1}{p'})}\Vert f\Vert _{L^p({\mathbb {R}}^2)}, \end{aligned}$$

where $\frac{1}{p}+\frac{1}{p'}=1$. This together with Theorem 2.13 and the intertwining property (1.9) gives the corresponding result for ${\mathcal {L}}_{{\textbf{A}},a}$:

#### Corollary 2.14

(Dispersive estimates of wave propagator). Suppose that ${\textbf{A}}(\theta )$ and $a(\theta )$ satisfy the assumptions of Theorem 2.13. Let $\varphi \in C_c^\infty ({\mathbb {R}}\setminus \{0\})$ with $0\le \varphi \le 1$ and $\text {supp}\;\varphi \subset [1/2,2]$. Then, for $1<p\le 2$,

$$\begin{aligned} \bigg \Vert \frac{\sin (t\sqrt{{\mathcal {L}}_{{\textbf{A}},a}})}{\sqrt{{\mathcal {L}}_{{\textbf{A}},a}}}\varphi (\sqrt{{\mathcal {L}}_{{\textbf{A}},a}})f\bigg \Vert _{L^{p'}({\mathbb {R}}^2)} \le C(1+|t|)^{-\frac{1}{2}(\frac{1}{p}-\frac{1}{p'})}\Vert f\Vert _{L^p({\mathbb {R}}^2)}. \end{aligned}$$

Recently, Fanelli–Zhang–Zheng [22] extended the uniform resolvent estimates for Laplacian to the pure magnetic potential. They proved that for all $p$ and $q$ satisfying

2.3 $$\begin{aligned} \frac{2}{3}\le \frac{1}{p}-\frac{1}{q}<1, \ \frac{3}{4}<\frac{1}{p}\le 1, \ \text {and} \ 0\le \frac{1}{q}<\frac{1}{4}, \end{aligned}$$

the following resolvent estimate is valid,

2.4 $$\begin{aligned} \left\| ({\mathcal {L}}_{{\textbf{A}},0}-z)^{-1}f\right\| _{L^{q} ({\mathbb {R}}^2)}\le C|z|^{\frac{1}{p}-\frac{1}{q}-1}\Vert f\Vert _{L^{p}({\mathbb {R}}^2)},\quad \text { for all }\;z\in {\mathbb {C}}\setminus {\mathbb {R}}^+. \end{aligned}$$

An immediate consequence of Theorem 2.13 and (2.4) is

#### Corollary 2.15

(Uniform resolvent estimate). Assume that ${\textbf{A}}(\theta )$ and $a(\theta )$ satisfy the assumptions of Theorem 2.13. Let (*p*, *q*) satisfy (2.3) and $1<p, q<\infty$. Then, we have

$$\begin{aligned} \left\| ({\mathcal {L}}_{{\textbf{A}},a}-z)^{-1}f\right\| _{L^{q} ({\mathbb {R}}^2)}\le C|z|^{\frac{1}{p}-\frac{1}{q}-1}\Vert f\Vert _{L^{p}({\mathbb {R}}^2)},\quad \text { for all }\;z\in {\mathbb {C}}\setminus {\mathbb {R}}^+. \end{aligned}$$

The Bochner–Riesz means of order $\delta$ associated with the any self-adjoint operator *H* defined using spectral theory by

$$\begin{aligned} S_R^\delta (H)=\Big (1-\frac{H}{R^2}\Big )^\delta _+, \end{aligned}$$

with $\delta \ge 0,\;R>0$. For the free Laplacian in dimension two, L. Carleson and P. Sjölin [9] proved that for $p \in [1,2)\cup (2,\infty ]$, the Bochner–Riesz means $S_R^\delta (-\Delta )f$ converges to *f* in $L^p({\mathbb {R}}^2)$ if and only if

$$\begin{aligned} \delta >\delta _c(p,2):=\max \Big \{0,2\Big |\frac{1}{2}-\frac{1}{p}\Big |-\frac{1}{2}\Big \}, \end{aligned}$$

and $\delta \ge 0$ when $p=2$. This result follows from the boundedness of $S^{\delta }_1(\Delta )$,

$$\begin{aligned} \Vert S^\delta _1(-\Delta )f\Vert _{L^p({\mathbb {R}}^2)}\le C\Vert f\Vert _{L^p({\mathbb {R}}^2)},\quad \delta >\delta _c(p,2). \end{aligned}$$

Recently, Miao–Yan–Zhang [35] extended this to the pure magnetic case, that is

$$\begin{aligned} \Vert S^\delta _1({\mathcal {L}}_{{\textbf{A}},0})f\Vert _{L^p({\mathbb {R}}^2)}\le C\Vert f\Vert _{L^p({\mathbb {R}}^2)},\quad \text { for all }\;1\le p\le \infty ,\;\delta >\delta (p,2). \end{aligned}$$

#### Corollary 2.16

($L^p$ boundedness for Bochner–Riesz means). Suppose that ${\textbf{A}}(\theta )$ and $a(\theta )$ satisfy the assumptions of Theorem 2.13. Let $\delta >\delta _c(p,2)$. Then, we have

$$\begin{aligned} \Vert S^\delta _1({\mathcal {L}}_{{\textbf{A}},a})f\Vert _{L^p({\mathbb {R}}^2)}\le C\Vert f\Vert _{L^p({\mathbb {R}}^2)},\quad \text { for all }\;1<p<\infty . \end{aligned}$$

As a consequence, we deduce that $S_R^\delta ({\mathcal {L}}_{{\textbf{A}},a})f$ converges to *f* in $L^p({\mathbb {R}}^2)$ as $\delta >\delta _c(p,2)$ and $1<p<\infty .$

## Preliminary Tools

In this section we give some preliminaries for the proof of Theorem 2.9. Let $\Gamma (z)=\int _0^\infty e^{-t} t^{z-1} \,\,\textrm{d}t$ be the Gamma function.

### Lemma 3.1

Let $a>0$ and $|b|\le 1$. Then,

3.1 $$\begin{aligned} \left| \frac{\Gamma (a+n+1)\Gamma (b+n+1)}{\Gamma (a+b+n+1) \Gamma (n+1)}\right| \le C a^{|b|},\;\quad \text { for all }\;n\in {\mathbb {N}}, \end{aligned}$$

as $a\rightarrow \infty$, where the constant *C* is independent of *a* and *n*.

### Proof

It follows (see [41]) from Stirling’s formula that $\big | \frac{\Gamma (a+n+1)}{\Gamma (a+b+n+1)}\big | \le C(a+n+1)^{-b}$ and $\big |\frac{\Gamma (b+n+1)}{\Gamma (n+1)}\big | \le C(n+1)^b$, which implies the result. $\square$

### Mellin Multipliers

Recall the Hankel transform ${\mathscr {H}}_\nu$ of order $\nu$ from (1.3). Given the definitions (1.5)–(1.7), we are led to consider the composition $T_{\nu ,\mu } :={\mathscr {H}}_{\nu } {\mathscr {H}}_{\mu }$. To study the $L^p$-boundedness of this operator, we will use the Mellin transform, defined by

$$\begin{aligned}{\mathcal {M}} (f(s))(z)=\int _0^{\infty } f(s)s^{z-1}\,\,\textrm{d}s,\end{aligned}$$

whenever the integral is absolutely convergent.

The Mellin transform turns the Hankel transform into multiplication by a certain ratio of Gamma functions. It follows that the operator $T_{\nu , \mu }$ is the Mellin multiplier

$$\begin{aligned} {\mathcal {M}} (T_{\nu , \mu } f)(z)=m_{\nu , \mu }(z){\mathcal {M}} ( f)(z) \end{aligned}$$

where $n(d)=\frac{d-2}{2}$ as in Theorem 2.9 and

$$\begin{aligned} m_{\nu , \mu }(z )=\frac{\Gamma (\frac{z+\nu -n(d)}{2}) \Gamma (1-\frac{z-\mu -n(d)}{2})}{\Gamma (1-\frac{z-\nu -n(d) }{2}) \Gamma (\frac{z+\mu -n(d)}{2})}. \end{aligned}$$

This leads to the following $L^p$ boundedness result:

#### Proposition 3.2

Let $\nu , \mu \ge 0$. The operator $T_{\nu , \mu }$ is bounded on $L^p({\mathbb {R}}^+,r^{d-1}\,\,\textrm{d}r)$ for all $p\in (1,\infty )$ such that ${n(d)-\nu }<\frac{d}{p}<{2+n(d)+\mu }$. In particular, if $\mu , \nu \ge \frac{d-2}{2}$, then $T_{\nu , \mu }$ is bounded on $L^p({\mathbb {R}}^+,r^{d-1}\,\,\textrm{d}r)$ for all $1<p<\infty$.

#### Proof

The result follows by taking^1^$\mu =-\frac{d}{p}$, $\alpha =n(d)-\nu$, and $\beta =2+n(d)+\mu$ in [33, Theorem 2.6] once we show:

(i) $m_{\nu , \mu }(z)$ is analytic in $\alpha<\mathop {\textrm{Re}}\limits z<\beta$,
(ii) $m_{\nu , \mu }(z)$ is bounded in $\alpha<\mathop {\textrm{Re}}\limits z<\beta$, and
(iii) for $\alpha<\mathop {\textrm{Re}}\limits z<\beta$, $|m'_{\nu , \mu }(z)| \le C_{\nu , \mu } |\mathop {\textrm{Im}}\limits z|^{-1}$ as $|\mathop {\textrm{Im}}\limits z|\rightarrow \infty$.

(ii) follows easily from (3.1), and (i) follows since $\Gamma (z)$ is analytic for $\mathop {\textrm{Re}}\limits z>0$, $1/\Gamma (z)$ is entire, and $\mathop {\textrm{Re}}\limits (1-\frac{z-\mu -n(d)}{2})> 0$ and $\mathop {\textrm{Re}}\limits \Gamma (\frac{z+\nu -n(d)}{2}) >0$ when $n(d)-\nu<\mathop {\textrm{Re}}\limits z<2+\mu +n(d)$.

As for (iii), by the formula $f'(z) =(\ln f(z))'f(z)$, one can get

$$\begin{aligned} m_{\nu , \mu }'(z)=\frac{1}{2} m_{\nu , \mu }(z)n_{\nu , \mu }(z) , \end{aligned}$$

where

$$\begin{aligned}  &   n_{\nu ,\mu }(z) =\psi \Big (\frac{z+\nu -n(d) }{2}\Big ) -\psi \Big (1-\frac{z-\mu -n(d)}{2}\Big )-\psi \Big (\frac{z+\mu -n(d) }{2}\Big ) \\  &   \quad \phantom {a} +\psi \big (1-\frac{z-\nu -n(d)}{2}\big ), \end{aligned}$$

and $\psi =\Gamma '/\Gamma$ is the digamma function, which has the asymptotic expansion

$$\begin{aligned} \psi (z)=\ln z-\frac{1}{2z}+O\left( \frac{1}{z^2}\right) , \quad \text {as}\quad |z| \rightarrow \infty , \end{aligned}$$

This gives the following asymptotic for $n_{\nu ,\mu }$:

$$\begin{aligned} n_{\nu , \mu }(z)&=\ln \left( 1+\frac{(\mu -\nu )(\mu +\nu +2)}{(z-n(d)+\mu )(z-n(d)-\mu -2)}\right) \\&\quad + \frac{2\nu +2}{(z-n(d)+\nu )(z-n(d)-\nu -2)}-\frac{2\mu +2}{(z-n(d)+\mu )(z-n(d)-\mu -2)} \\&\quad +O\left( \frac{1}{|z|^2}\right) . \end{aligned}$$

Thus, $|n_{\nu , \mu }(z)|\le C_{\nu , \mu }/|z|$ as $|z| \rightarrow \infty .$ Using this with the boundedness of $m_{\nu , \mu }(z)$, we see that (iii) is satisfied, and the proof is completed. $\square$

### Singular Integral Operators on the Half-line

Following the proof of [34, Lemma 2.11], we obtain

#### Lemma 3.3

Let $\mu \ge 0$ and $\nu \ge 0$. For $r\in {\mathbb {R}}^{+}$, define

$$\begin{aligned} Tf(r)&=\int _\frac{r}{2}^r \left( \frac{s}{r}\right) ^{\mu +1+\frac{d}{2}-\frac{d}{p}}\frac{ f(s)}{1-(\frac{s}{r})^2} \frac{\,\,\textrm{d}s}{s} -\int _r^{2r} \left( \frac{r}{s}\right) ^{\frac{d}{p}-\frac{d}{2}+1+\nu }\frac{f(s)}{1-(\frac{r}{s})^2} \frac{\,\,\textrm{d}s}{s}, \end{aligned}$$

then there exists a constant $C=C(\mu ,\nu )>0$ such that

$$\begin{aligned} \Vert Tf\Vert _{L^p({\mathbb {R}}^{+}, \frac{\,\,\textrm{d}r}{r})}\le C\Vert f\Vert _{L^p({\mathbb {R}}^{+}, \frac{\,\,\textrm{d}r}{r})},\quad \text { for all }\;1<p<\infty . \end{aligned}$$

## Proof of Main Theorem

To prove Theorem 2.9, note that it suffices to show that for any $p\in (1,\infty )$ satisfying $\frac{n(d)-\widetilde{\nu }_1}{d}\le \frac{1}{p}\le \frac{1}{2}$, one has

$$\begin{aligned} \Vert Wf\Vert _{L^{p}({\mathbb {R}}^d)}\le C\Vert f\Vert _{L^p({\mathbb {R}}^d)}. \end{aligned}$$

This is because *W* and $W^*$ can be treated in the same way: if we exchange the roles of $\mu _{\alpha }$ with $\nu _{\alpha }$, and $e_{\alpha }$ with $\phi _{\alpha }$, an entirely analogous argument applied to $W^*$ shows that $W^*$ is also $L^p({\mathbb {R}}^d)$-bounded for any $p\in (1,\infty )$ satisfying $\frac{n(d)-\widetilde{\mu }_1}{d}\le \frac{1}{p}\le \frac{1}{2}$. Then Theorem 2.9 follows immediately by duality. So in the remainder of the paper, we will only consider $p$ in this range.

Since $L_{{\textbf{A}},a}$ is a proper perturbation of $L_{{\textbf{A}},0}$, when $j\ge \ell$,

4.1 $$\begin{aligned} |\widetilde{\mu }_{jk}-\widetilde{\nu }_{jk}|=\frac{|\widetilde{\mu }_{jk}^2-\widetilde{\nu }_{jk}^2|}{\widetilde{\mu }_{jk}+\widetilde{\nu }_{jk}} \le \frac{C}{j } \quad \text {and}\quad \phi _{jk}(\theta )=e_{jk}(\theta )+e_{jk}(\theta ) R_{jk}(\theta ), \end{aligned}$$

with

$$\begin{aligned} \sup _{1\le k\le m_j} \Vert R_{jk}(\theta )\Vert _{L^\infty ({\mathbb {S}}^{d-1})} <\frac{C}{j }, \end{aligned}$$

where $$\widetilde{\mu }_{\smash {jk}}^{2}=\sqrt{\mu _{jk}^{2}+n(d)^2}$$, $$\widetilde{\nu }_{\smash {jk}}^{2}=\sqrt{\nu _{jk}^{2}+n(d)^2}$$.

Without loss of generality, assume that $\ell$ is so large that when $j \ge \ell$, we have

4.2 $$\begin{aligned} \widetilde{\mu }_{jk}, \widetilde{\nu }_{jk} \ge \frac{d-2}{2}+C j, \ \text {for all} \ 1\le k\le m_j, \end{aligned}$$

where *C* is a small constant.

Using (4.1), we split $\Vert Wf\Vert _{L^p({\mathbb {R}}^d)}$ into three terms:

$$\begin{aligned} \Vert Wf\Vert _{L^{p}({\mathbb {R}}^{d})}&=\bigg \Vert \sum _{\alpha \in {\mathcal {I}}_{\ell }^{\perp }\cup {\mathcal {I}}_\ell } W_{\alpha }^r (f_{\alpha }(\cdot )\big )(r)W_{\alpha }^\theta \big (e_{\alpha }(\theta ))\bigg \Vert _{L^p} \\&\lesssim \sum _{i=1}^{\ell _0}\left\| {\mathscr {H}}_{\widetilde{\nu }_i}{\mathscr {H}}_{\widetilde{\mu }_i}f_{i}(r)\phi _{i}(\theta )\right\| _{L^p} +\bigg \Vert \sum _{j,k} {\mathscr {H}}_{\widetilde{\nu }_{jk}}{\mathscr {H}}_{\widetilde{\mu }_{jk}}f_{jk}(r) e_{jk}(\theta ) \bigg \Vert _{L^p} \\&\quad +\bigg \Vert \sum _{j,k} {\mathscr {H}}_{\widetilde{\nu }_{jk}}{\mathscr {H}}_{\widetilde{\mu }_{jk}}f_{jk}(r)R_{jk}(\theta ) e_{jk}(\theta ) \bigg \Vert _{L^p}=:I_1 + I_2 + I_3, \end{aligned}$$

where $$\widetilde{\mu }_i=\sqrt{\mu _i^2+n(d)^{2}}$$ and $$\widetilde{\nu }_i=\sqrt{\nu _i^2+n(d)^{2}}$$.

We will next show that $I_1$, $I_2$, and $I_3$ are each bounded by $\Vert f\Vert _{L^p}$.

### The estimate of $I_1$

Let $\max \{0, \frac{n(d)-\widetilde{\nu }_1}{d}\}\le \frac{1}{p}\le \frac{1}{2}$. For each fixed $\alpha \in {\mathcal {I}}_{\ell }^{\perp }$, it follows from [20, Lemma 2.1] that

$$\begin{aligned} \Vert \phi _{\alpha }(\theta )\Vert _{L^\infty ({\mathbb {S}}^{d-1})}\lesssim |\nu _\alpha |^d. \end{aligned}$$

This implies that

$$\begin{aligned} \left\| W_{\alpha }^rf_{\alpha }(r)\phi _{\alpha }(\theta )\right\| _{L^p({\mathbb {R}}^d)}&\lesssim _\alpha \left\| W_{\alpha }^rf_{\alpha }(r)\right\| _{L^p(r^{d-1}\,\,\textrm{d}r)} \nonumber \\&\lesssim _\alpha \Vert f_{\alpha }(r)\Vert _{L^p(r^{d-1}\,\,\textrm{d}r)}, \end{aligned}$$

where the last inequality follows from Proposition 3.2, since $\widetilde{\nu }_{\alpha } \ge \widetilde{\nu }_1 \ge 0$. By Hölder’s inequality, we get for $p\ge 2$

4.3 $$\begin{aligned} \Vert f_{\alpha }(r)\Vert _{L^{p}(r^{d-1}\,\,\textrm{d}r)}&\lesssim \bigg \Vert \bigg ( \sum _{\alpha }|f_{\alpha }(r)|^{2} \bigg )^{1/2} \bigg \Vert _{L^{p}(r^{d-1}\,\,\textrm{d}r)} \nonumber \\&= \bigg \Vert \sum _{\alpha }f_{\alpha }(r)e_{\alpha }(\theta ) \bigg \Vert _{L^p(r^{d-1} \,\,\textrm{d}r ; L^{2}(d\theta ))} \nonumber \\&\lesssim \bigg \Vert \sum _{\alpha }f_{\alpha }(r)e_{\alpha }(\theta ) \bigg \Vert _{L^{p}(r^{d-1}\,\,\textrm{d}rd\theta )} =\Vert f\Vert _{L^{p}({\mathbb {R}}^{d})}. \end{aligned}$$

Hence,

$$\begin{aligned} I_1\lesssim \sum _{i=1}^{\ell _0}C_i \Vert f_i\Vert _{L^{p}({\mathbb {R}}^{d})}\lesssim _\ell \Vert f\Vert _{L^{p}({\mathbb {R}}^{d})}. \end{aligned}$$

### The estimate of $I_3$

Since $L_{{\textbf{A}},a}$ is a proper perturbation of $L_{{\textbf{A}},0}$, by the assumption that $\{e_{\alpha }\}_{\alpha }\in \mathcal {DMB}$, we immediately get that $I_3\lesssim I_2.$

### The estimate of $I_2$

This is given by the following proposition.

#### Proposition 4.1

Assume that the eigenbasis $\{e_\alpha \}_{\alpha \in {\mathcal {I}}_{\ell }^{\perp }\cup {\mathcal {I}}_\ell }$ of $L_{{\textbf{A}},0}$ belongs to $\mathcal {DMB}$. Then for all $1<p<\infty$,

4.4 $$\begin{aligned} \!\!\!\!\!\!\!\! I_2 = \bigg \Vert \sum _{j = \ell }^{\infty }\sum _{k=1}^{m_j} {\mathscr {H}}_{\widetilde{\nu }_{jk}}{\mathscr {H}}_{\widetilde{\mu }_{jk}}f_{jk}(r) e_{jk}(\theta ) \bigg \Vert _{L^p({\mathbb {R}}^{d})}\lesssim \bigg \Vert \sum _{j =\ell }^\infty \sum _{k=1}^{m_j} f_{jk}(r)e_{jk}(\theta )\bigg \Vert _{L^p({\mathbb {R}}^{d})}. \end{aligned}$$

#### Proof

This is similar to the proof for the main theorem in [34] so we have put it in Appendix A for the interested reader. $\square$

Using Proposition 4.1 and (4.3), we obtain

$$\begin{aligned} I_2&\lesssim \bigg \Vert \sum _{j=\ell }^\infty \sum _{k=1}^{m_j} f_{jk}e_{jk}\bigg \Vert _{L^p} \lesssim \bigg \Vert \sum _{\alpha \in {\mathcal {I}}_{\ell }^{\perp }\cup {\mathcal {I}}_\ell }f_{\alpha }e_{\alpha }\bigg \Vert _{L^p} + \sum _{\alpha \in {\mathcal {I}}_{\ell }^{\perp }}\left\| f_{\alpha }e_{\alpha }\right\| _{L^p} \lesssim _\ell \Vert f\Vert _{L^p}. \end{aligned}$$

This completes the proof of Theorem 2.9. $\square$

## The Proof of Theorem 2.13

Assume that $a\in W^{1,\infty }({\mathbb {S}}^{1},{\mathbb {R}})$, ${\textbf{A}}\in W^{1,\infty }({\mathbb {S}}^{1},{\mathbb {R}}^2)$ through this section. We will prove Theorem 2.13 using Theorem 2.9. To do this, we need to verify Assumption 1.1, show that $\{e_\alpha \}_{\alpha \in {\mathcal {I}}_{\ell }^{\perp }\cup {\mathcal {I}}_\ell } \in \mathcal {DMB}$, and prove that $L_{{\textbf{A}}, a}$ is a proper perturbation of $L_{{\textbf{A}}, 0}$. We split the proof into the following cases:

1. $${\widetilde{A}}\notin \frac{1}{2}{\mathbb {Z}}$$,
2. ${\widetilde{A}}\in {\mathbb {Z}}$ and *a* is symmetric across $\pi$, or
3. ${\widetilde{A}}\in \frac{1}{2}{\mathbb {Z}}\setminus {\mathbb {Z}}$ and *a* is symmetric across $\pi$.

In the proof, we will need properties of the *T*-periodic eigenvalue problem.

### Definition 5.1

For each $a \in W^{1,\infty }({\mathbb {S}}^1, {\mathbb {R}})$ and $T\in (-1/2,1/2]$, the *T*-*periodic eigenvalue problem* is the following ODE,

5.1 $$\begin{aligned} {\left\{ \begin{array}{ll} -\psi ''(\theta )+a(\theta )\psi (\theta ) =\lambda \psi (\theta ),\quad \theta \in [0,2\pi ] \\ \psi (2\pi )=e^{2i\pi T}\psi (0), \quad \psi '(2\pi )= e^{2\pi i T}\psi '(0), \end{array}\right. } \end{aligned}$$

It admits an increasing sequence $\{\lambda _n(T,a)\}_{n\ge 1}$ of eigenvalues $\lambda _n(T,a)\uparrow \infty$, and the following comparison principle from [12, Theorem 2.2.2]:

### Lemma 5.2

Let $a,b\in W^{1,\infty }$ and $T\in {\mathbb {R}}$. If $a \ge b$ pointwise, then for every $n\ge 1$,

$$\begin{aligned} \lambda _n(T,a) \ge \lambda _n(T,b). \end{aligned}$$

### When ${\widetilde{A}} \notin \frac{1}{2}{\mathbb {Z}}$

Let ${\bar{A}}$ be the number in $(-1/2,1/2)\setminus \{0\}$ such that ${\bar{A}} - {\widetilde{A}} \in {\mathbb {Z}}$. We will only consider ${\bar{A}} \in (0,\frac{1}{2})$, as the case ${\bar{A}}<0$ can be treated analogously after adjusting notation (for example, we would need to exchange $\mu _{j1}^2$ and $\mu _{j2}^2$ below to match the order of eigenvalues in Assumption 1.1(1)). From [12, Section 2.3], we know that the free spherical operator $L_{{\textbf{A}},0}=(-i\partial _\theta +A(\theta ))^2$ with periodic boundary condition has eigenvalues

$$\begin{aligned} \mu _0^2={\bar{A}}^2, \quad \mu _{jk}^2 = (j +(-1)^{k}{\bar{A}})^2,\ \text {for all} \ \ j\ge 1, k=1, 2. \end{aligned}$$

The corresponding cluster sizes are $m_0=1$ and $m_j=2$ for $j\ge 1$, and the corresponding eigenfunctions are

$$\begin{aligned} e_0(\theta )=\frac{1}{\sqrt{2\pi }}e^{i({\bar{A}}\theta - \int _0^\theta A(\theta ') {\,\,\textrm{d}{\theta }}')},\qquad e_{jk}(\theta )=\frac{1}{\sqrt{2\pi }}e^{ij (-1)^{k}\theta }e^{i({\bar{A}}\theta - \int _0^\theta A(\theta ') {\,\,\textrm{d}{\theta }}')}. \end{aligned}$$

Clearly, $$\{e_{jk}(\theta )\} \in \mathcal {DMB}$$ since $$\{e_0(\theta ), \frac{1}{\sqrt{2\pi }}e^{ij \theta }, \frac{1}{\sqrt{2\pi }}e^{-ij \theta }\}_{j \ge 1}\in \mathcal {DMB}$$.

Let $\{\lambda _n({\textbf{A}},a)\}_{n\ge 1}$ be the eigenvalues of $L_{{\textbf{A}}, a}$ arranged as an increasing sequence diverging to infinity. From the proof of [17, Lemma 2.1], we know that there exist $n^*,\ell \in {\mathbb {N}}$ such that $\lambda _n({\textbf{A}},a)$ can be written as

$$\begin{aligned}\{\lambda _n({\textbf{A}}, a):\;n\in {\mathbb {N}},\;n>n^*\}=\{\nu _{jk}^2:\;j\ge \ell , k=1,2\},\end{aligned}$$

with

5.2 $$\begin{aligned} \nu _{jk}^2={\widetilde{a}}+\mu _{jk}^2+O\Big (\frac{1}{j^2}\Big ),\ \text {as} \ j\rightarrow \infty . \end{aligned}$$

By taking $n^*$ and $\ell$ sufficiently large, we may assume for later use that

5.3 $$\begin{aligned} |\nu _{jk}^2-{\widetilde{a}}-\mu _{jk}^2|< 1, \text { for all } j\ge \ell .\end{aligned}$$

It follows from (5.2) that $L_{{\textbf{A}},0}$ and $L_{{\textbf{A}}, a}$ satisfy Assumption 1.1 (1) with any choice of $C_{{\textbf{A}}} \in (0, \frac{1}{2} - {\bar{A}})$.

Next, we will show that $L_{{\textbf{A}},0}$ and $L_{{\textbf{A}}, a}$ satisfy Assumption 1.1 (2). Starting from the eigenvalue equation,

$$\begin{aligned} {\left\{ \begin{array}{ll} L_{{\textbf{A}},a}\phi _n(\theta )=\lambda _n({\textbf{A}},a)\phi _n(\theta ),\quad \theta \in [0,2\pi ] \\ \phi _n(0)=\phi _n(2\pi ),\;\phi _n'(0)=\phi _n'(2\pi ), \end{array}\right. } \end{aligned}$$

we can use the gauge transformation

$$\begin{aligned} \psi (\theta ) = e^{-i\int _0^\theta A(\theta '){\,\,\textrm{d}{\theta }}'}\phi (\theta ), \end{aligned}$$

to obtain the ${\bar{A}}$-periodic eigenvalue problem (5.1), with $\lambda _n({\textbf{A}},a) = \lambda _n({\bar{A}},a)$.

For *j* sufficiently large, the eigenvalues $\lambda _{2j}({\bar{A}}, a)$ and $\lambda _{2j+1}({\bar{A}}, a)$ are equal to $\nu ^2_{j'k'}$ for some $j',k'$, and the goal now is to show that we can identify $j'$ and $k'$ for $\lambda _{2\ell }({\bar{A}}, a)$ and $\lambda _{2\ell +1}({\bar{A}}, a)$. Notice that $(\mathop {\textrm{span}}\limits _{j\ge \ell }\{e_{jk}\})^\perp$ is spanned by the $2\ell -1$ vectors $$\{e_0\}\cup \{e_{jk}:\begin{array}{c} j=1,2,\dots ,\ell ,\\ k=1,2 \end{array}\}$$. So we need to show that $n^*=2\ell -1$.

Let $a_*=-\Vert a\Vert _{L^\infty }-1$ and $a^*=\Vert a\Vert _{L^\infty }+1$. By the comparison principle, we have that for all $n\ge 0$,

$$\begin{aligned} \lambda _{n}({\bar{A}}, a_*)<\lambda _{n}({\bar{A}}, a)<\lambda _{n}({\bar{A}}, a^*). \end{aligned}$$

Since $\lambda _{2j}({\bar{A}},0) = \mu _{j1}^2$ and $\lambda _{2j+1}({\bar{A}},0)=\mu _{j2}^2$, we have

$$\begin{aligned} \mu _{j1}^2+a_*<\lambda _{2j}({\bar{A}}, a)<\mu _{j1}^2+a^*, {\text { and}} \\ \mu _{j2}^2+a_*<\lambda _{2j+1}({\bar{A}}, a)<\mu _{j2}^2+a^*. \end{aligned}$$

Define for $j\ge \ell$ the intervals $I_j=[\mu _{j1}^2+a_*, \mu _{j2}^2+a^*]$, which contain $\lambda _{2j}({\bar{A}},a)$ and $\lambda _{2j+1}({\bar{A}},a)$. By taking $\ell \gg 1$ if necessary, the $I_j$ are pairwise disjoint. Note that by (5.3), $I_j$ contains precisely two eigenvalues

$$\begin{aligned} \{\nu ^2_{j'k'}: j'\ge \ell ,\ k'=1,2 \}\cap I_j = \{ \nu _{j1}^2,\ \nu _{j 2}^2\}. \end{aligned}$$

Hence, $\nu _{j1}^2=\lambda _{2j}$ and $\nu _{j2}^2=\lambda _{2j+1}$ for $j\ge \ell$. In particular, for $j=\ell$, we have $n^*=2\ell -1$. This proves that $L_{{\textbf{A}},0}$ and $L_{{\textbf{A}}, a}$ satisfy Assumption 1.1 (2) with $\ell _0=2\ell -1$.

Now we check the proper perturbation property. By [17, Lemma 2.1], the corresponding normalized eigenfunctions $\phi _{jk}$ can be written as

$$\begin{aligned} \phi _{jk}(\theta )=e_{jk}(\theta )\big (1+R_{jk}(\theta )\big ) \end{aligned}$$

with

$$\begin{aligned} R_{jk}(\theta ) = e^{(-1)^{k} i( \sqrt{\nu _{\smash {jk}}^2 - {\widetilde{a}}} - \mu _{jk} )\theta } e^{i \int _0^\theta W_{jk}(\theta ') {\,\,\textrm{d}{\theta }}' } - 1 \end{aligned}$$

where $$W_{jk}$$ satisfies

5.4 $$\begin{aligned} \big \Vert W_{jk}\big \Vert _{C^0({\mathbb {S}}^{1})}\le \frac{C}{\sqrt{\nu _{\smash {jk}}^2-{\widetilde{a}}}}\le \frac{C}{j },\quad \text {as}\quad j \rightarrow \infty . \end{aligned}$$

A simple calculation gives

$$\begin{aligned} R'_{jk} = (-1)^{k}i(R_{jk}(\theta ) + 1 ) \big (\sqrt{\nu _{\smash {jk}}^2 - {\widetilde{a}}} - \mu _{jk} + W_{jk}(\theta )\big ). \end{aligned}$$

By (5.2) and (5.4), we get for any $\theta \in [0,2\pi ]$ and $k=1, 2$

$$\begin{aligned} |R_{jk}(\theta )|\le \frac{C}{j },\quad |R_{jk}'(\theta )|\le \frac{C}{j },\quad \text {as}\quad j \rightarrow \infty . \end{aligned}$$

Hence,

$$\begin{aligned} \sup _{\begin{array}{c} j \ge \ell \\ \theta \in [0,2\pi ] \end{array}} |R_{j1}(\theta )|+|R_{j2}(\theta )|+|R_{j1}'(\theta )|+|R_{j2}'(\theta )|\le \frac{C}{\ell }, \end{aligned}$$

and

$$\begin{aligned} \sup _{\begin{array}{c} j \ge \ell \\ \theta \in [0,2\pi ] \end{array}} \sum _{j =2^L}^{2^{L+1}} \Big ( |{\mathfrak {D}}R_{j1}'(\theta )|+|{\mathfrak {D}}R_{j2}'(\theta )|\Big )\le \frac{C}{\ell }. \end{aligned}$$

In conclusion, $L_{{\textbf{A}}, a}$ is a proper perturbation of $L_{{\textbf{A}}, 0}$.

The conditions for Theorem 2.9 have been verified, and we deduce that the wave operator *W* and $W^*$ are bounded on $L^p({\mathbb {R}}^2)$ for $1<p<\infty$ due to $\mu _1,\nu _1\ge 0.$ This proves Theorem 2.13 when ${\widetilde{A}}\not \in \frac{1}{2}{\mathbb {Z}}.$

### When ${\widetilde{A}}\in {\mathbb {Z}}$ and *a* is Symmetric Across $\pi$

In this case, the eigenvalues and eigenfunctions of $L_{{\textbf{A}},0}$ are given by

$$\begin{aligned} \mu _0^2&=0,&e_0(\theta )&=\frac{1}{\sqrt{2\pi }}, \\ \mu _{j1}^2&=j^2,&e_{j1}(\theta )&=\frac{1}{\sqrt{\pi }}e^{-i\int _0^{\theta }A(\theta ')\,\,\textrm{d}\theta '} \sin j\theta , \\ \mu _{j2}^2&= j^2,&e_{j2}(\theta )&=\frac{1}{\sqrt{\pi }}e^{-i\int _0^{\theta }A(\theta ')\,\,\textrm{d}\theta '} \cos j\theta . \end{aligned}$$

For the perturbed operator $L_{{\textbf{A}},a}\varphi =(-i\partial _\theta +A(\theta ))^2\varphi +a(\theta )\varphi$, by [17, Lemma B.7], there exists $\ell \in {\mathbb {N}}$ such that $\{\lambda _n:\; n \in {\mathbb {N}},\;n \ge n^*\}=\{\nu _{jk}^2: j \ge \ell ,\;k=1,2\}$ and

$$\begin{aligned} \nu _{jk}^2=\mu _{jk}^2+{\widetilde{a}}+O\Big (\frac{1}{j }\Big ),\quad \text {as}\quad j \rightarrow \infty . \end{aligned}$$

Using the comparison principle as before, one can prove that $n^*=2\ell -1$, so $L_{{\textbf{A}}, 0}$ and $L_{{\textbf{A}}, a}$ satisfy the Assumption 1.1.

Under the symmetry assumption, *a* is given by a cosine series. Let $a_{{{\,\textrm{c}\,}}, k}$ denote the $k^\text {th}$ cosine-Fourier coefficient of *a*(*x*) and write

$$\begin{aligned} R_{jk,{{\,\textrm{s}\,}}}(\theta )=&\frac{1}{2}\sum _{m\ne j}\frac{a_{{{\,\textrm{c}\,}},|j-m|}+(-1)^k a_{{{\,\textrm{c}\,}},j+m}}{(j-m)(j+m)}\sin ((m-j)\theta ), \\ R_{jk,{{\,\textrm{c}\,}}}(\theta )=&\frac{1}{2}\sum _{m\ne j}\frac{a_{{{\,\textrm{c}\,}},|j-m|}+(-1)^k a_{c,j+m}}{(j-m)(j+m)}\cos ((m-j)\theta ). \end{aligned}$$

Direct computation gives

$$\begin{aligned} \sup _{\theta }\{|R_{jk,{{\,\textrm{s}\,}}}(\theta )|, |R_{jk,{{\,\textrm{c}\,}}}(\theta )|, |R_{jk,{{\,\textrm{s}\,}}}'(\theta )|, |R_{jk,{{\,\textrm{c}\,}}}'(\theta )|\}\lesssim \frac{1}{j}. \end{aligned}$$

Then the corresponding eigenfunctions $\phi _{jk}$ can be written as

$$\begin{aligned} \phi _{j1}(\theta )=e_{j1}(\theta )\big (1+R_{j1, {{\,\textrm{c}\,}}}(\theta )+R_{j1}^2(\theta )\big )+ e_{j2}(\theta ) R_{j1, {{\,\textrm{s}\,}}}(\theta ), \\ \phi _{j2}(\theta )=e_{j2}(\theta )\big (1+R_{j2, {{\,\textrm{c}\,}}}(\theta )+R_{j2}^2(\theta )\big )+ e_{j1}(\theta ) R_{j2, {{\,\textrm{s}\,}}}(\theta ), \end{aligned}$$

where

$$\begin{aligned} \Vert R_{jk}^2\Vert _{L^\infty }= O\Big (\frac{1}{j^2}\Big ),\quad \text {as}\;j \rightarrow \infty . \end{aligned}$$

Strictly speaking, the remainder term does not satisfy Assumption (2) in Definition 2.4, as we do not know the estimate for the derivatives of $R_{jk}^2$. That is, $L_{{\textbf{A}},a }$ is a proper perturbation of $L_{{\textbf{A}}, 0}$ with a negligible term.

However, this term has enough decay for us to use the much weaker triangle inequality instead of the discrete multiplier theorem to get the $L^p$-boundedness. Here are the details: we only need to handle the term induced by $R_{jk}^2$. For this term, we have

$$\begin{aligned}&\bigg \Vert \sum _{\begin{array}{c} j\ge \ell \\ k=1,2 \end{array}} W_{jk}^rf_{jk}(r)R_{jk}^2(\theta ) e_{jk}(\theta ) \bigg \Vert _{L^p({\mathbb {R}}^{2})} \lesssim \sum _{j,k} \frac{1}{j^2} \left\| W_{jk}^rf_{jk}(r) \right\| _{L^p(r\;dr)} \\&\lesssim \sup _{j\ge \ell ,k} \left\| W_{jk}^rf_{jk}(r) \right\| _{L^p(r\;dr)} \lesssim \bigg \Vert \sum _{j,k} W_{jk}^rf_{jk}(r)e_{jk}(\theta ) \bigg \Vert _{L^p({\mathbb {R}}^{2})} \\&\lesssim \bigg \Vert \sum _{j,k} f_{jk}(r)e_{jk}(\theta ) \bigg \Vert _{L^p({\mathbb {R}}^{2})} \lesssim \Vert f\Vert _{L^p({\mathbb {R}}^2)}. \end{aligned}$$

Therefore, Theorem 2.13 is proved when ${\widetilde{A}}\in {\mathbb {Z}}$ and $a(\theta )$ is symmetric across $\theta =\pi$.

### When ${\widetilde{A}}\in \frac{1}{2}{\mathbb {Z}}\setminus {\mathbb {Z}}$ and *a* is Symmetric Across $\pi$

In this case, the operator $L_{{\textbf{A}}, 0}$ has the following eigenbasis

$$\begin{aligned}  &   \mu _{j1}^2=\mu _{j2}^2 =\Big (j+\frac{1}{2}\Big )^2, \ j\ge 0 \\  &   \quad e_{j1} =\frac{1}{\sqrt{2\pi }} e^{i\int _0^\theta A(s)\,\,\textrm{d}s} \sin \Big (\Big (j+\frac{1}{2}\Big ) \theta \Big ), \quad e_{j2}=\frac{1}{\sqrt{2\pi }}e^{i\int _0^\theta A(s)\,\,\textrm{d}s} \cos \Big (\Big (j+\frac{1}{2}\Big ) \theta \Big ). \end{aligned}$$

To state the properties of the eigenfunctions for the perturbed operator, we write

$$\begin{aligned} {\widehat{a}}(\theta )={\left\{ \begin{array}{ll} 4a(2\theta ), &  \text {if}\ \theta \in [0, \pi ], \\ 4a(2\theta -2\pi ), &  \text {if}\ \theta \in [\pi , 2\pi ]. \end{array}\right. } \end{aligned}$$

Let ${\widehat{a}}_{{{\,\textrm{c}\,}}, k}$ denote the $k^\text {th}$ cosine-Fourier coefficient of ${\widehat{a}}(x)$ and write

$$\begin{aligned} {\widehat{R}}_{jk,{{\,\textrm{s}\,}}}(\theta )=&\frac{1}{2}\sum _{m\ne 2j+1}\frac{{\widehat{a}}_{{{\,\textrm{c}\,}},|2j+1-m|}+(-1)^k {\widehat{a}}_{{{\,\textrm{c}\,}},2j+1+m}}{(2j+1-m)(2j+1+m)}\sin ((m-2j-1)\theta /2), \\ {\widehat{R}}_{jk,{{\,\textrm{c}\,}}}(\theta )=&\frac{1}{2}\sum _{m\ne 2j+1}\frac{{\widehat{a}}_{{{\,\textrm{c}\,}},|2j+1-m|}+(-1)^k {\widehat{a}}_{{{\,\textrm{c}\,}},2j+1+m}}{(2j+1-m)(2j+1+m)}\cos ((m-2j-1)\theta /2). \end{aligned}$$

Similarly as before, the following estimates are valid,

$$\begin{aligned} \sup _{\theta }\{|{\widehat{R}}_{jk,{{\,\textrm{s}\,}}}(\theta )|, |{\widehat{R}}_{jk,{{\,\textrm{s}\,}}}(\theta )| |{\widehat{R}}_{jk,{{\,\textrm{s}\,}}}'(\theta )|, |{\widehat{R}}_{jk,{{\,\textrm{s}\,}}}'(\theta )|\}\lesssim \frac{1}{j}. \end{aligned}$$

For the perturbed operator $L_{{\textbf{A}},a}=(-i\partial _\theta +A(\theta ))^2+a(\theta )$, from the proof of [17, Lemma B.10], and [17, Lemma B.7], we deduce that there exists $\ell \in {\mathbb {N}}$ such that $\{\lambda _n:n\in {\mathbb {N}},\;n>2\ell \}=\{\nu _{j, k}^2:j\in {\mathbb {Z}},\;j \ge \ell ,\; k=1,2\}$ and

$$\begin{aligned} \nu _{j, k}^2={\widetilde{a}}+\Big (j+\frac{1}{2}\Big )^2+O\Big (\frac{1}{j }\Big ),\quad \text {as}\quad j \rightarrow \infty . \end{aligned}$$

The corresponding eigenfunctions $\phi _{j, k}$ satisfy

$$\begin{aligned} \phi _{j1}(\theta )=e_{j1}(\theta )\big (1+{\widehat{R}}_{j1, c}^1(\theta )+{\widehat{R}}_{j1}^2(\theta )\big )+ e_{j2}(\theta ) {\widehat{R}}_{j1, s}(\theta ), \\ \phi _{j2}(\theta )=e_{j2}(\theta )\big (1+{\widehat{R}}_{j2, c}^1(\theta )+{\widehat{R}}_{j2}^2(\theta )\big )+ e_{j1}(\theta ){\widehat{R}}_{j2, s}(\theta ), \end{aligned}$$

where

$$\begin{aligned} \Vert {\widehat{R}}_{jk}^2\Vert _{L^\infty }= O\Big (\frac{1}{j^2}\Big ),\quad \text {as}\;j \rightarrow \infty . \end{aligned}$$

As before, we have that *W* and $W^*$ are bounded in $L^p$. This concludes the final case and the proof of Theorem 2.13 is complete.$\square$

## Appendix A. Proof of Proposition 4.1

For any function *f*(*x*) with the expansion

$$\begin{aligned}f(r, \theta )=\sum _{j,k} f_{jk}(r)e_{jk}(\theta ).\end{aligned}$$

Define the operator $W_\ell$ by

$$\begin{aligned} W_\ell f(r,\theta )=\sum _{j = \ell }^{\infty }\sum _{k=1}^{m_j}{\mathscr {H}}_{\widetilde{\nu }_{jk}}{\mathscr {H}}_{\widetilde{\mu }_{jk}}f_{jk}(r) e_{jk}(\theta ). \end{aligned}$$

Then, we rewrite $W_\ell$ as

$$\begin{aligned} W_\ell f(r,\theta )&=\sum _{j,k}\int _0^\infty f_{jk}(s)\left( \int _0^\infty J_{\widetilde{\nu }_{jk}} (r\lambda )J_{\widetilde{\mu }_{jk}} (s\lambda ) \lambda ^{d-1}\,\,\textrm{d}{\lambda }\right) s\,\,\textrm{d}s e_{jk}(\theta ) \\&=\sum _{j,k} \int _0^\infty f_{jk}(s) K_{jk}(r,s)\frac{ds}{s}e_{jk}(\theta ), \end{aligned}$$

with the kernel

$$\begin{aligned}K_{jk}(r,s)=s^d \int _0^\infty J_{\widetilde{\nu }_{jk}} (r\lambda )J_{\widetilde{\mu }_{jk}} (s\lambda ) \lambda ^{d-1}\,\,\textrm{d}{\lambda }.\end{aligned}$$

Hence, (4.4) is equivalent to

$$\begin{aligned} \Vert W_\ell f\Vert _{L^p({\mathbb {R}}^d)}\le C\Vert f\Vert _{L^p({\mathbb {R}}^d)}, \end{aligned}$$

which can also be written as

A.1 $$\begin{aligned} \Vert r^{\frac{d}{p}} W_\ell f\Vert _{L^p(\frac{\,\,\textrm{d}r}{r} \cdot {\,\,\textrm{d}{\theta }})}\le C\Vert r^{\frac{d}{p}} f\Vert _{L^p(\frac{\,\,\textrm{d}r}{r} \cdot {\,\,\textrm{d}{\theta }})}. \end{aligned}$$

So we define the modified kernels

$$\begin{aligned} {\widetilde{K}}_{jk}(r,s)=\left( \frac{s}{r}\right) ^{-\frac{d}{p}}K_{jk}(r,s)=\left( \frac{s}{r}\right) ^{-\frac{d}{p}}s^d \int _0^\infty J_{\widetilde{\nu }_{jk}} (r\lambda )J_{\widetilde{\mu }_{jk}} (s\lambda ) \lambda ^{d-1}\,\,\textrm{d}{\lambda }, \end{aligned}$$

and the corresponding modified wave operator

$$\begin{aligned} {\widetilde{W}}_\ell f(r,\theta )=\sum _{j =\ell }^{\infty }\sum _{k=1}^{m_j}\int _0^\infty {\widetilde{K}}_{jk}(r,s)f_{jk}(s)\frac{\,\,\textrm{d}s}{s}e_{jk}(\theta ). \end{aligned}$$

Then, (A.1) can be reduced to prove

A.2 $$\begin{aligned} \big \Vert {\widetilde{W}}_\ell f(r,\theta )\big \Vert _{L^p(\frac{\,\,\textrm{d}r}{r} \cdot {\,\,\textrm{d}{\theta }})}\le C\Vert f(r, \theta )\Vert _{L^p(\frac{\,\,\textrm{d}r}{r} \cdot {\,\,\textrm{d}{\theta }})}. \end{aligned}$$

Let

$$\begin{aligned} a_{jk}&=\frac{\widetilde{\mu }_{jk}+\widetilde{\nu }_{jk}}{2},\; b_{jk}=\frac{\widetilde{\mu }_{jk}-\widetilde{\nu }_{jk}}{2}. \end{aligned}$$

Since $L_{{\textbf{A}},0}$ and $L_{{\textbf{A}},a}$ satisfies the Assumption 1.1, and ${\mathcal {L}}_{{\textbf{A}}, a}$ is a proper perturbation of ${\mathcal {L}}_{{\textbf{A}}, 0}$, one has

A.3 $$\begin{aligned} |a_{jk}|\simeq j ,\quad | b_{jk}|\le \frac{1}{j }, \quad a_{jk} b_{jk}=O(1), \ \text {for}\ j \ge \ell . \end{aligned}$$

By [34, (4.9)], we get the explicit formula for the kernel functions ${\widetilde{K}}_{jk}(r, s)$ as follows:

$$\begin{aligned} {\widetilde{K}}_{jk}(r, s)=&\frac{s^{\frac{d}{2}+1-\frac{d}{p}}}{r^{\frac{d}{2}-1-\frac{d}{p}}}\int _0^\infty \lambda J_{\widetilde{\mu }_{jk}}(s\lambda ) J_{\widetilde{\nu }_{jk}}(r \lambda ) \,\,\textrm{d}{\lambda } \nonumber \\&={\left\{ \begin{array}{ll} 2\left( \frac{s}{r}\right) ^{\frac{d}{2}+1-\frac{d}{p}+\widetilde{\mu }_{jk}} \frac{\sin (-\pi b_{jk})}{\pi } \sum _{n=0}^\infty A_{j,k, n}^{+}\left( \frac{s}{r}\right) ^{2n}, &  0<s<r; \\ 2\left( \frac{r}{s}\right) ^{{\frac{d}{p}-\frac{d}{2}+1+\widetilde{\nu }_{jk}}} \frac{\sin (\pi b_{jk})}{\pi } \sum _{n=0}^\infty A_{j,k, n}^{-}\left( \frac{r}{s}\right) ^{2n}, &  0<r<s, \end{array}\right. } \end{aligned}$$

with

$$\begin{aligned} A_{j,k, n}^{+}&=\frac{\Gamma (a_{jk}+n+1)\Gamma (b_{jk}+n+1)}{\Gamma (a_{jk}+b_{jk}+n+1) \Gamma (n+1)}, \\ A_{j,k, n}^{-}&= \frac{\Gamma (a_{jk}+n+1)\Gamma (1- b_{jk}+n)}{\Gamma (a_{jk}- b_{jk}+n+1) \Gamma (n+1)}. \end{aligned}$$

The asymptotic behavior of ${\widetilde{K}}_{jk}(r,s)$ suggests that we should split ${\widetilde{W}}_\ell$ into the three parts as follows:

$$\begin{aligned} {\widetilde{W}}_\ell f(r, \theta )&=\int _{0}^{\infty } \sum _{j,k} {\widetilde{K}}_j(r,s) f_{jk}(s) \frac{\,\,\textrm{d}s}{s}e_{jk}(\theta ) \\&=\int _{0}^{\frac{r}{2}} \sum _{j,k} {\widetilde{K}}_j(r,s) f_{jk}(s) \frac{\,\,\textrm{d}s}{s}e_{jk}(\theta )+\int _{\frac{r}{2}}^{2r} \sum _{j,k} {\widetilde{K}}_j(r,s) f_{jk}(s) \frac{\,\,\textrm{d}s}{s}e_{jk}(\theta ) \\&\quad +\int _{2r}^{\infty } \sum _{j,k} {\widetilde{K}}_j(r,s) f_{jk}(s) \frac{\,\,\textrm{d}s}{s}e_{jk}(\theta ) \\&=:{\widetilde{W}}_{\ell ,1} f(r, \theta )+{\widetilde{W}}_{\ell ,2} f(r, \theta )+{\widetilde{W}}_{\ell ,3} f(r, \theta ). \end{aligned}$$

Then, showing (A.2) is reduced to proving that

A.4 $$\begin{aligned} \Vert {\widetilde{W}}_{\ell ,k} f(r, \theta )\Vert _{L^p(\frac{\,\,\textrm{d}r}{r} \cdot {\,\,\textrm{d}{\theta }})} \lesssim \Vert f(r, \theta )\Vert _{L^p(\frac{\,\,\textrm{d}r}{r} \cdot {\,\,\textrm{d}{\theta }})}, \ \text {for}\ k= 1, 2, 3. \end{aligned}$$

We define ${\widetilde{K}}_{j,k, 1}(r,s)$, ${\widetilde{K}}_{j,k, 2}(r,s)$ and ${\widetilde{K}}_{j,k, 3}(r,s)$ by

$$\begin{aligned} {\widetilde{K}}_{j,k, 1}(r,s)&= {\widetilde{K}}_{jk}(r,s) \chi _{\{0<s<r/2\}}, \\ {\widetilde{K}}_{j,k, 2}(r,s)&= {\widetilde{K}}_{jk}(r,s) \chi _{\{r/2<s<2r\}}, \\ {\widetilde{K}}_{j,k, 3}(r,s)&= {\widetilde{K}}_{jk}(r,s) \chi _{\{2r<s\}}. \end{aligned}$$

### Case 1: $0<\frac{s}{r}<\frac{1}{2}$.

Recall

$$\begin{aligned}{\widetilde{K}}_{j,k, 1}(r,s)=2\left( \frac{s}{r}\right) ^{\frac{d}{2}+1-\frac{d}{p}+\widetilde{\mu }_{jk}} \frac{\sin (-\pi b_{jk})}{\pi } \sum _{n=0}^\infty A_{j,k, n}^{+}\left( \frac{s}{r}\right) ^{2n}\chi _{\{0<s<r/2\}}\end{aligned}$$

and

$$\begin{aligned}A_{j,k, n}^{+}=\frac{\Gamma (a_{jk}+n+1)\Gamma (b_{jk}+n+1)}{\Gamma (a_{jk}+b_{jk}+n+1) \Gamma (n+1)}.\end{aligned}$$

By (A.3) , and using Lemma 3.1 with $a=a_{jk}\simeq j$ and $b= b_{jk},$ we deduce that

$$\begin{aligned} \big |A_{j,k, n}^{+}\big |\le Cj . \end{aligned}$$

And so for $1\le k \le m_j$, by (4.2), one has

A.5 $$\begin{aligned} |{\widetilde{K}}_{j,k, 1}(r,s)|\lesssim \left( \frac{s}{r}\right) ^{\frac{d}{2}+1-\frac{d}{p}+\widetilde{\mu }_{jk}} j \lesssim \left( \frac{s}{r}\right) ^{d -\frac{d}{p}} \frac{j }{2^{|Cj|}}\lesssim \left( \frac{s}{r}\right) ^{d -\frac{d}{p}}, \end{aligned}$$

where the above constant is independent of *j*. Hence, for the average $\overline{{\widetilde{K}}}_{j, 1}(r,s)$ of ${\widetilde{K}}_{j,k, 1}(r,s)$, one has

$$\begin{aligned} \sup _{j}|\overline{{\widetilde{K}}}_{j, 1}(r,s)| \lesssim \left( \frac{s}{r}\right) ^{d -\frac{d}{p}}. \end{aligned}$$

On the other hand,

$$\begin{aligned} \sup _{L\in {\mathbb {N}}:\;2^L\ge \ell } 2^{(N-1)L} \sum _{j =2^L}^{2 ^{L+1}}\left| {\mathfrak {D}}^N\overline{{\widetilde{K}}}_{j, 1}(r,s)\right|&\lesssim \sum _{j =\ell }^{\infty } j ^{N-1}\sup _{1\le k \le m_j}| {\widetilde{K}}_{j,k, 1}(r,s)| \nonumber \\&\lesssim \sum _{j =\ell }^{\infty } j ^N \sup _{1\le k \le m_j}\left( \frac{s}{r}\right) ^{\frac{d}{2}+1-\frac{d}{p}+\widetilde{\mu }_{jk}}\nonumber \\&\lesssim \left( \frac{s}{r}\right) ^{d-\frac{d}{p}}. \end{aligned}$$

This inequality together with (A.5) shows that ${\widetilde{K}}_{j,k, 1}(r,s)$ satisfies the conditions (2.1) and (2.2). Since $\{e_{jk}(\theta )\}_j \in \mathcal {DMB}$, then for $1<p<\infty$, we have

$$\begin{aligned} \left\| \sum _{j,k}{\widetilde{K}}_{j,k, 1}(r,s) f_{jk}(s) e_{jk}(\theta ) \right\| _{L^{p}({\,\,\textrm{d}{\theta }})} \lesssim \left( \frac{s}{r}\right) ^{d-\frac{d}{p}}\left\| \sum _{j,k} f_{jk}(s) e_{jk}(\theta )\right\| _{L^{p}({\,\,\textrm{d}{\theta }})}. \end{aligned}$$

Furthermore, notice $r ^{d-\frac{d}{p}}\chi _{\{0<r<1/2\}} \in L^1(\frac{\,\,\textrm{d}r}{r})$ for all $1<p<\infty$. Combining this fact and log-Young’s inequality, we have

A.6 $$\begin{aligned} \Vert {\widetilde{W}}_{\ell ,1} f(r, \theta )\Vert _{ L^p (\frac{\,\,\textrm{d}r}{r} \cdot {\,\,\textrm{d}{\theta }})}&\lesssim \left\| \int _0^{r/2} \left( \frac{s}{r}^{}\right) ^{d-\frac{d}{p}} \left\| \sum _{j,k} f_{jk}(s) e_{jk}(\theta )\right\| _{L^{p}({\,\,\textrm{d}{\theta }})} \frac{\,\,\textrm{d}s}{s} \right\| _{L^p (\frac{\,\,\textrm{d}r}{r})}\nonumber \\&\lesssim \left\| \sum _{j,k} f_{jk}(s)e_{jk}(\theta )\right\| _{L^p(\frac{\,\,\textrm{d}r}{r} \cdot {\,\,\textrm{d}{\theta }})} =\Vert f(r,\theta )\Vert _{L^{p}(\frac{\,\,\textrm{d}r}{r} \cdot {\,\,\textrm{d}{\theta }})}. \end{aligned}$$

### Case 2: $\frac{s}{r}>2$.

Recall

$$\begin{aligned}{\widetilde{K}}_{j,k, 3}(r,s)=2\left( \frac{r}{s}\right) ^{{\frac{d}{p}-\frac{d}{2}+1+\widetilde{\nu }_{jk}}} \frac{\sin (\pi b_{jk})}{\pi } \sum _{n=0}^\infty A_{j,k, n}^{-}\left( \frac{r}{s}\right) ^{2n}\chi _{\{0<r<s/2\}}\end{aligned}$$

and

$$\begin{aligned}A_{j,k, n}^{-}= \frac{\Gamma (a_{jk}+n+1)\Gamma (1- b_{jk}+n)}{\Gamma (a_{jk}- b_{jk}+n+1) \Gamma (n+1)}.\end{aligned}$$

By (A.3), and using Lemma 3.1 with $a=a_{jk}\simeq j$ and $b=- b_{jk},$ we obtain

$$\begin{aligned}\big |A_{j,k, n}^{-}\big |\le Cj .\end{aligned}$$

And so

A.7 $$\begin{aligned} |{\widetilde{K}}_{j,k, 3}( r, s)|\lesssim \left( \frac{r}{s}\right) ^{\frac{d}{p}+Cj} j \lesssim \left( \frac{r}{s}\right) ^{\frac{d}{p}}\frac{j }{2^{Cj }} \lesssim \left( \frac{r}{s}\right) ^{\frac{d}{p}}, \end{aligned}$$

and

A.8 $$\begin{aligned} \sup _{L\in {\mathbb {N}}: 2^L\ge \ell } 2^{(N-1)L}\sum _{j=2^L}^{2 ^{L+1}}\left| {\mathfrak {D}}^N \overline{{\widetilde{K}}}_{j, 3}(r,s)\right|&\lesssim \sum _{j =\ell }^{\infty } j^{N-1}\sup _{1\le k \le m_j} | {\widetilde{K}}_{j,k, 3}(r,s)| \nonumber \\&\lesssim \sum _{j =\ell }^{\infty } j^N \left( \frac{r}{s}\right) ^{\frac{d}{p}+Cj} \lesssim \left( \frac{r}{s}\right) ^{\frac{d}{p}}. \end{aligned}$$

From (A.7) and (A.8), we know that ${\widetilde{K}}_{j,k, 3}(r,s)$ satisfies the condition (2.1) and (2.2), which implies the following bound is valid:

$$\begin{aligned} \left\| \sum _{j,k} {\widetilde{K}}_{j,k, 3}(r,s) f_{jk}(s) e_{jk}(\theta ) \right\| _{L^{p}({\,\,\textrm{d}{\theta }})} \lesssim \left( \frac{r}{s}\right) ^{\frac{d}{p}}\left\| \sum _{j,k} f_{jk}(s) e_{jk}(\theta ) \right\| _{L^{p}({\,\,\textrm{d}{\theta }})}. \end{aligned}$$

Noting that $r^{\frac{d}{p}}\chi _{\{0<r<1/2\}}\in L^1(\frac{\,\,\textrm{d}r}{r})$ for $1<p<\infty$, we get

A.9 $$\begin{aligned} \Vert {\widetilde{W}}_{\ell ,3} f(r, \theta )\Vert _{ L^p (\frac{\,\,\textrm{d}r}{r}\cdot {\,\,\textrm{d}{\theta }})}&\lesssim \Bigg \Vert \int _{2r}^{\infty } \left( \frac{r}{s}\right) ^{\frac{d}{p}} \bigg \Vert \sum _{j,k} f_{jk}(s) e_{jk}(\theta ) \bigg \Vert _{L^{p}({\,\,\textrm{d}{\theta }})} \frac{\,\,\textrm{d}s}{s} \Bigg \Vert _{L^p (\frac{\,\,\textrm{d}r}{r})} \nonumber \\&\lesssim \bigg \Vert \sum _{j,k} f_{jk}(s)e_{jk}(\theta ) \bigg \Vert _{L^p (\frac{\,\,\textrm{d}r}{r}\cdot {\,\,\textrm{d}{\theta }})}=\Vert f(r,\theta )\Vert _{L^p (\frac{\,\,\textrm{d}r}{r}\cdot {\,\,\textrm{d}{\theta }})}. \end{aligned}$$

Hence, ${{\widetilde{W}}}_{\ell ,3}$ is bounded in $L^p (\frac{\,\,\textrm{d}r}{r} \cdot {\,\,\textrm{d}{\theta }})$. And so, (A.4) with $k=3$ follows.

For the rest part of this section, we remain to show that ${{\widetilde{W}}}_{\ell , 2}$ is bounded in $L^p (\frac{\,\,\textrm{d}r}{r} \cdot {\,\,\textrm{d}{\theta }})$.

### Case 3: $\frac{1}{2}<\frac{s}{r}<2$

Now we deal with the term ${\widetilde{W}}_{\ell ,2} f(r, \theta )$. Here we use similar strategy as in [34] and we define an approximate kernel ${\widetilde{k}}^{\text {ap}}(r,s)$ with the property that the index *k* and variables *r*, *s* are separated, and that they also have the same singularity as ${\widetilde{K}}_{jk}(r,s)$. For the approximating operator, we use the oscillatory in the radial part first. After that, we use the multiplier theorem for the eigenfunctions to get the boundedness for the angular part. For the error term, we use the oscillatory in the angular part first, then using Young’s inequality to control the radial part.

In the remainder of this section, we use the shorthand notation $\chi _{+}=\chi _{\{r/2<s<r\}}$ and $\chi _{-}=\chi _{\{r<s<2r\}}$. Sometimes we omit $\chi _{+}$ and $\chi _{-}$ when we have already restricted the range of *r*, *s* appropriately.

Now we are in position to prove the following inequality

$$\begin{aligned} \Vert {\widetilde{W}}_{\ell ,2} f(r, \theta )\Vert _{L^p(\frac{\,\,\textrm{d}r}{r} \cdot {\,\,\textrm{d}{\theta }})} \lesssim \Vert f(r, \theta )\Vert _{L^p(\frac{\,\,\textrm{d}r}{r} \cdot {\,\,\textrm{d}{\theta }})}. \end{aligned}$$

Using Stirling’s formula, we have for any fixed *j*, as *n* goes to $\infty$,

$$\begin{aligned} A_{j,k, n}^{+}&=1-\frac{a_{jk} b_{jk}}{n+1}+O_j\left( \frac{1}{(n+1)^2}\right) , \\ A_{j,k, n}^{-}&=1+\frac{a_{jk} b_{jk}}{(n+1)}+O_j\left( \frac{1}{(n+1)^2}\right) . \end{aligned}$$

This inspired us to rewrite ${\widetilde{K}}_{j,k, 2}$ as

$$\begin{aligned} {\widetilde{K}}_{j,k, 2}&= 2\left( \frac{s}{r}\right) ^{\frac{d}{2}+1-\frac{d}{p}+\widetilde{\mu }_{jk}} \frac{\sin (-\pi b_{jk})}{\pi } \left[ \frac{1}{1-(\frac{s}{r})^2}-a_{jk} b_{jk}\left( \frac{s}{r}\right) ^{-2} \ln \!\Big ({1-\Big (\frac{s}{r}\Big )^2}\Big ) +E_{jk}^{+}\right] \chi _+ \\&\quad +2\left( \frac{r}{s}\right) ^{{\frac{d}{p}-\frac{d}{2}+1+\widetilde{\nu }_{jk}}} \frac{\sin (\pi b_{jk})}{\pi } \left[ \frac{1}{1-(\frac{r}{s})^2} +a_{jk} b_{jk}\left( \frac{r}{s}\right) ^{-2} \ln \!\Big ({1-\Big (\frac{r}{s}\Big )^2}\Big ) +E_{jk}^{-} \right] \chi _- \\&=: {\widetilde{K}}_{j,k, 2}^1 (r,s)+{\widetilde{K}}_{j,k, 2}^2 (r,s)+{\widetilde{K}}_{j,k, 2}^3 (r,s) \end{aligned}$$

with

$$\begin{aligned} {\widetilde{K}}_{j,k, 2}^1(r,s)&=2\left( \frac{s}{r}\right) ^{\frac{d}{2}+1-\frac{d}{p}+\widetilde{\mu }_{jk}} \frac{\sin (-\pi b_{jk})}{\pi }\frac{1}{1-\left( \frac{s}{r}\right) ^2}\chi _+ \\&\quad +2\left( \frac{r}{s}\right) ^{{\frac{d}{p}-\frac{d}{2}+1+\widetilde{\nu }_{jk}}} \frac{\sin (\pi b_{jk})}{\pi }\frac{1}{1-(\frac{r}{s})^2}\chi _-; \\ {\widetilde{K}}_{j,k, 2}^2 (r,s)&=-2a_{jk} b_{jk} \left( \frac{s}{r}\right) ^{\frac{d}{2}-1-\frac{d}{p}+\widetilde{\mu }_{jk}} \frac{\sin (- \pi b_{jk})}{\pi }\ln \!\Big ({1-\Big (\frac{s}{r}\Big )^2}\Big ) \chi _+ \\&\quad +2a_{jk} b_{jk} \left( \frac{r}{s}\right) ^{{\frac{d}{p}-\frac{d}{2}-1+\widetilde{\nu }_{jk}}} \frac{\sin (\pi b_{jk})}{\pi }\ln \!\Big ({1-\Big (\frac{r}{s}\Big )^2}\Big ) \chi _-; \\ {\widetilde{K}}_{j, k,2}^3 (r,s)&=2 \left( \frac{s}{r}\right) ^{\!\frac{d}{2}+1-\frac{d}{p}+\widetilde{\mu }_{jk}} \frac{\sin (- \pi b_{jk})}{\pi } E_{jk}^+(r,s) \\&\quad +2 \left( \frac{r}{s}\right) ^{\!{\frac{d}{p}-\frac{d}{2}+1+\widetilde{\nu }_{jk}}} \frac{\sin (\pi b_{jk})}{\pi }E_{jk}^-(r,s). \end{aligned}$$

We denote the corresponding operators ${\widetilde{W}}_{\ell ,2}^1, {\widetilde{W}}_{\ell ,2}^2$ and ${\widetilde{W}}_{\ell ,2}^3$ by

$$\begin{aligned} {\widetilde{W}}_{\ell ,2}^{i} f(r, \theta )=\int _0^\infty \sum _{j,k}{\widetilde{K}}_{j,k, 2}^i(r,s) f_{jk}(s)e_{jk}(\theta )\frac{\,\,\textrm{d}s}{s}, \qquad i=1, 2, 3. \end{aligned}$$

***Estimate for the first term ***${\widetilde{W}}_{\ell ,2}^{1}$.

Step 1: *Estimate for the approximate kernels: *Let us define the approximate kernels by

$$\begin{aligned} {\widetilde{K}}_{j,k, 2}^{1, \text {ap}}(r,s)&= 2 \left( \frac{s}{r}\right) ^{\frac{d}{2}+1-\frac{d}{p}+\widetilde{\mu }_{\ell , 1}} \frac{\sin (-\pi b_{jk})}{\pi }\frac{1}{1-\left( \frac{s}{r}\right) ^2}\chi _+ \\  &\quad + 2 \left( \frac{r}{s}\right) ^{\frac{d}{p}-\frac{d}{2}+1+\widetilde{\nu }_{\ell , 1}} \frac{\sin \pi b_{jk}}{\pi }\frac{1}{1-(\frac{r}{s})^2}\chi _-, \end{aligned}$$

and the corresponding operator ${\widetilde{W}}_{\ell ,2}^{1, \text {ap}}f (r, \theta )$ by

$$\begin{aligned}&{\widetilde{W}}_{\ell ,2}^{1, \text {ap}}f(r,\theta )\ :=\sum _{j,k} \int _{0}^\infty {\widetilde{K}}_{j,k, 2}^{1, \text {ap}}(r,s) f_{jk}(s)\frac{\,\,\textrm{d}s}{s}e_{jk}(\theta ) \\&\quad =2\int _\frac{r}{2}^r \left( \frac{s}{r}\right) ^{\frac{d}{2}+1-\frac{d}{p}+\widetilde{\mu }_{\ell , 1}}\frac{1}{1-(\frac{s}{r})^2} \sum _{j,k} \frac{\sin (-\pi b_{jk})}{\pi } f_{jk}(s)e_{jk}(\theta )\frac{\,\,\textrm{d}s}{s} \\&\quad +2\int _{r}^{2r} \left( \frac{r}{s}\right) ^{\frac{d}{p}-\frac{d}{2}+1+\widetilde{\nu }_{\ell , 1}}\frac{1}{1-(\frac{r}{s})^2} \sum _{j,k}\frac{\sin \pi b_{jk}}{\pi } f_{jk}(s)e_{jk}(\theta )\frac{\,\,\textrm{d}s}{s}. \end{aligned}$$

Using Lemma 3.3, we have

$$\begin{aligned} \Vert {\widetilde{W}}_{\ell ,2}^{1, \text {ap}}f(r,\theta )\Vert _{L^p(\frac{dr}{r})}\lesssim \bigg \Vert \sum _{j,k} \sin \pi b_{jk} f_{jk}(r)e_{jk}(\theta )\bigg \Vert _{L^p(\frac{dr}{r})}. \end{aligned}$$

We can easily check that the sequence $\{\sin \pi b_{jk}\}$ satisfies the condition (2.1) and (2.2). Hence

A.10 $$\begin{aligned} \Vert {\widetilde{W}}_{\ell ,2}^{1, \text {ap}}f(r,\theta )\Vert _{L^p(\frac{dr}{r}\cdot d\theta )}\lesssim \bigg \Vert \sum _{j,k} f_{jk}(r)e_{jk}(\theta )\bigg \Vert _{L^p(\frac{dr}{r}\cdot d\theta )}. \end{aligned}$$

Step 2:

* Estimate for the error term*. Define the kernels of the error terms

$$\begin{aligned} {\widetilde{K}}_{j,k, 2}^{1, \text {err}}(r,s)&={\widetilde{K}}_{j,k, 2}^{1}(r,s)-{\widetilde{K}}_{j,k, 2}^{1, \text {ap}}(r,s) \\&=2\left( \frac{s}{r}\right) ^{\frac{d}{2}+1-\frac{d}{p}+\widetilde{\mu }_{\ell , 1}} \frac{\sin (-\pi b_{jk})}{\pi }\frac{1}{1-\left( \frac{s}{r}\right) ^2}\left[ \left( \frac{s}{r}\right) ^{{\widetilde{\mu }_{jk}-\widetilde{\mu }_{\ell , 1}}}-1\right] \chi _+ \\&\quad +2\left( \frac{r}{s}\right) ^{\frac{d}{p}-\frac{d}{2}+1+\widetilde{\nu }_{\ell , 1}} \frac{\sin (\pi b_{jk})}{\pi }\frac{1}{1-(\frac{r}{s})^2}\left[ \left( \frac{r}{s}\right) ^{{\widetilde{\nu }_{jk}-\widetilde{\nu }_{\ell , 1}}}-1\right] \chi _-, \end{aligned}$$

and the associated operator

$$\begin{aligned} {\widetilde{W}}_{\ell ,2}^{1, \text {err}} f(r, \theta )=\int _0^\infty \sum _{j,k}{\widetilde{K}}_{j,k, 2}^{1, \text {err}}(r,s) f_{jk}(s)e_{jk} (\theta )\frac{\,\,\textrm{d}s}{s}. \end{aligned}$$

By the same argument as in [34, Lemma 4.3, Lemma 4.5], we have

$$\begin{aligned} | \overline{{\widetilde{K}}}_{j 2}^{1, \text {err}}(r,s)|\lesssim 1, \quad \sup _{L\in {\mathbb {N}}:2^L\ge \ell } 2^{(N-1)L} \sum _{j=2^L}^{2^{L+1}}|{\mathfrak {D}}^N \overline{{\widetilde{K}}}_{j, 2}^{1, \text {err}}(r,s)|\lesssim 1. \end{aligned}$$

Hence

$$\begin{aligned} \Vert {\widetilde{W}}_{\ell ,2}^{1, \text {err}} f(r, \theta ) \Vert _{L^p({\,\,\textrm{d}{\theta }})} \le \int _{r/2}^r \Big \Vert \sum _{j,k}f_{jk}(s)e_{jk}(\theta )\Big \Vert _{L^p({\,\,\textrm{d}{\theta }})}\frac{\,\,\textrm{d}s}{s}. \end{aligned}$$

Furthermore, we obtain

A.11 $$\begin{aligned} \Vert {\widetilde{W}}_{\ell ,2}^{1, \text {err}} f(r, \theta )\Vert _{L^p(\frac{dr}{r}\cdot \,\,\textrm{d}\theta )} \le \Vert f(r, \theta )\Vert _{L^p(\frac{dr}{r}\cdot \,\,\textrm{d}\theta )}. \end{aligned}$$

Combining this with (A.10), we get the $L^p$-boundedness of ${\widetilde{W}}_{\ell ,2}^1$.

***Estimate for the second term*** ${\widetilde{W}}_{\ell ,2}^{2}$. Direct computation gives

$$\begin{aligned} | \overline{{\widetilde{K}}_{j, 2}} (r,s)|&\lesssim \left| \ln \!\Big ({1-\Big (\frac{s}{r}\Big )^2}\Big )\right| \chi _++\left| \ln \!\Big ({1-\Big (\frac{r}{s}\Big )^2}\Big )\right| \chi _-, \\ \sup _L 2^{(N-1)L} \sum _{j=2^L}^{2^{L+1}}|{\mathfrak {D}}^N \overline{{\widetilde{K}}_{j, 2}^2}(r,s)|&\lesssim \left| \ln \!\Big ({1-\Big (\frac{s}{r}\Big )^2}\Big )\right| \chi _++\left| \ln \!\Big ({1-\Big (\frac{r}{s}\Big )^2}\Big )\right| \chi _-. \end{aligned}$$

Since $\{e_\alpha \}_{\alpha \in {\mathcal {I}}_{\ell }^{\perp }\cup {\mathcal {I}}_\ell } \in \mathcal {DMB}$, we have

$$\begin{aligned} \Vert {\widetilde{W}}_{\ell ,2}^{2} f(r, \theta ) \Vert _{L^p({\,\,\textrm{d}{\theta }})}&\le \int _{r/2}^r \left| \ln \!\Big ({1-\Big (\frac{s}{r}\Big )^2}\Big ) \right| \bigg \Vert \sum _{j,k} f_{jk}(s) e_{jk}(\theta ) \bigg \Vert _{L^p({\,\,\textrm{d}{\theta }})} \frac{\,\,\textrm{d}s}{s} \\&\quad +\int _r^{2r}\left| \ln \!\Big ({1-\Big (\frac{r}{s}\Big )^2}\Big )\right| \bigg \Vert \sum _{j,k} f_{jk}(s)e_{jk}(\theta ) \bigg \Vert _{L^p({\,\,\textrm{d}{\theta }})}\frac{\,\,\textrm{d}s}{s}. \end{aligned}$$

Hence, by Young’s inequality, we get

A.12 $$\begin{aligned} \Vert {\widetilde{W}}_{\ell ,2}^{2} f(r, \theta ) \Vert _{L^p(\frac{\,\,\textrm{d}r}{r} \cdot \,\,\textrm{d}{\theta })} \lesssim \Vert f(r, \theta )\Vert _{L^p(\frac{\,\,\textrm{d}r}{r} \cdot \,\,\textrm{d}{\theta })}. \end{aligned}$$

***Estimate for the third term*** ${\widetilde{W}}_{\ell ,2}^{3}$. Recall that ${\widetilde{K}}_{j,k, 2}^3$ is given by

$$\begin{aligned} {\widetilde{K}}_{j,k, 2}^3 (r,s)&= 2 \left( \frac{s}{r}\right) ^{\!\frac{d}{2}+1-\frac{d}{p}+\widetilde{\mu }_{jk}}\frac{\sin (-\pi b_{jk})}{\pi }\sum _{n=0}^{\infty } E_{j,k, n}^{+}\left( \frac{s}{r}\right) ^{2n}\chi _+ \\&\quad +2 \left( \frac{r}{s}\right) ^{\frac{d}{p}-\frac{d}{2}+1+\widetilde{\nu }_{jk}} \frac{\sin \pi b_{jk}}{\pi }\sum _{n=0}^{\infty }E_{j,k,n}^{-}\left( \frac{r}{s}\right) ^{2n} \chi _-,\end{aligned}$$

where

$$\begin{aligned} E_{j,k, n}^{+}= A_{j,k, n}^{+}-1+\frac{a_{jk} b_{jk}}{n+1},\ 1\le k \le m_j ; \\ E_{j,k, n}^{-}= A_{j,k, n}^{-}-1-\frac{a_{jk} b_{jk}}{n+1},\ 1\le k \le m_j. \end{aligned}$$

Follow the same argument as in [34, Lemma 4.6], we can prove that

$$\begin{aligned} \bigg | \frac{\,\,\textrm{d}^N E_{j,k, n}^{+}}{\,\,\textrm{d}j^N}\bigg | \lesssim \frac{1}{j^N} \frac{1}{n+1}, \\ \bigg |\frac{\,\,\textrm{d}^N E_{j,k, n}^{-}}{\,\,\textrm{d}j^N}\bigg | \lesssim \frac{1}{j^N} \frac{1}{n+1} . \end{aligned}$$

These will lead to that ${\widetilde{K}}_{j, 2}^3 (r,s)$ will satisfies the conditions (2.1) and (2.2) with an upper bound $|\ln \left( 1-(\frac{s}{r})^2\right) |$ for fixed *r*, *s*, hence

$$\begin{aligned} \Vert {\widetilde{W}}_{\ell ,2}^{3} f(r, \theta ) \Vert _{L^p({\,\,\textrm{d}{\theta }})}&\le \int _{r/2}^r \Big |\ln \!\Big ({1-\Big (\frac{s}{r}\Big )^2}\Big )\Big | \Big \Vert \sum _{j,k}f_{jk}(s)e_{jk}(\theta )\Big \Vert _{L^p({\,\,\textrm{d}{\theta }})}\frac{\,\,\textrm{d}s}{s} \\&+\int _r^{2r}\Big |\ln \!\Big ({1-\Big (\frac{r}{s}\Big )^2}\Big )\Big | \Big \Vert \sum _{j,k}f_{jk}(s)e_{jk}(\theta )\Big \Vert _{L^p({\,\,\textrm{d}{\theta }})}\frac{\,\,\textrm{d}s}{s}. \end{aligned}$$

Log-Young’s inequality again gives

A.13 $$\begin{aligned} \Vert {\widetilde{W}}_{\ell ,2}^{3} f(r, \theta ) \Vert _{L^p(\frac{\,\,\textrm{d}r}{r} \cdot \,\,\textrm{d}{\theta })} \lesssim \Vert f(r, \theta )\Vert _{L^p(\frac{\,\,\textrm{d}r}{r} \cdot \,\,\textrm{d}{\theta })}. \end{aligned}$$

Combining (A.11), (A.12) and (A.13), we have proved that

$$\begin{aligned} \Vert {\widetilde{W}}_{\ell ,2} f(r,\theta )\Vert _{L^p(\frac{\,\,\textrm{d}r}{r} \cdot \,\,\textrm{d}{\theta })} \lesssim \Vert f(r, \theta )\Vert _{L^p(\frac{\,\,\textrm{d}r}{r} \cdot \,\,\textrm{d}{\theta })}. \end{aligned}$$

This inequality together with (A.6) and (A.9) implies that ${\widetilde{W}}$ is bounded in $L^p(\frac{\,\,\textrm{d}r}{r} \cdot \,\,\textrm{d}{\theta })$ for $1<p<\infty$. Hence, we conclude the proof Proposition 4.1.

## Footnotes

1 This $\mu$ is not the same $\mu$ as in our proposition.
